# The zinc-finger protein Red1 orchestrates MTREC submodules and binds the Mtl1 helicase arch domain

**DOI:** 10.1038/s41467-021-23565-3

**Published:** 2021-06-08

**Authors:** Nikolay Dobrev, Yasar Luqman Ahmed, Anusree Sivadas, Komal Soni, Tamás Fischer, Irmgard Sinning

**Affiliations:** 1 Heidelberg University Biochemistry Center (BZH), Heidelberg, Germany; 2 The John Curtin School of Medical Research, The Australian National University, Canberra, ACT Australia

**Keywords:** Proteins, X-ray crystallography

## Abstract

Cryptic unstable transcripts (CUTs) are rapidly degraded by the nuclear exosome in a process requiring the RNA helicase Mtr4 and specific adaptor complexes for RNA substrate recognition. The PAXT and MTREC complexes have recently been identified as homologous exosome adaptors in human and fission yeast, respectively. The eleven-subunit MTREC comprises the zinc-finger protein Red1 and the Mtr4 homologue Mtl1. Here, we use yeast two-hybrid and pull-down assays to derive a detailed interaction map. We show that Red1 bridges MTREC submodules and serves as the central scaffold. In the crystal structure of a minimal Mtl1/Red1 complex an unstructured region adjacent to the Red1 zinc-finger domain binds to both the Mtl1 KOW domain and stalk helices. This interaction extends the canonical interface seen in Mtr4-adaptor complexes. In vivo mutational analysis shows that this interface is essential for cell survival. Our results add to Mtr4 versatility and provide mechanistic insights into the MTREC complex.

## Abstract (web summary)

The human PAXT complex and the MTREC complex in fission yeast are important exosome cofactors, serving in the degradation of specific noncoding RNAs. Here, the authors combine structural, biochemical and in vivo methods to show how Red1 recruits the Mtl1 helicase by an interface not seen before in helicase-adaptor complexes.

## Introduction

Genome-wide pervasive transcription in eukaryotic cells results in a considerable amount of non-coding RNA (ncRNA) transcripts, most of which are rapidly degraded by the nuclear RNA surveillance machinery. The nuclear exosome is a central player in this process, but the mechanism of how the exosome can selectively degrade pervasive transcripts and distinguish them from mRNAs is not well understood. The exosome is an evolutionarily conserved multi-subunit complex found in the cytosol and in the nucleus, and it is essential for cell viability^1,2^. It comprises a catalytically inactive core composed of nine subunits, resembling a barrel-like structure^1,3^. Ribonuclease activity is provided by the 3′–5′ exonuclease Rrp6 (EXOSC10 in human)^1,4^ and/or 3′–5′ exo-/endonuclease Rrp44 (Dis3 or EXOSC11 in human)^1^. The exosome only processes single-stranded RNAs and its activity requires a helicase^5^. In the cytosol, helicase activity is provided by Ski2, while in the nucleus it is provided by Mtr4p/hMTR4 (refs. ^6,7^). The Mtr4–exosome interaction is facilitated by Rrp6/Rrp47 (refs. ^8,9^), and a recent cryo-EM structure revealed that the protein Mpp6 also provides a stable tether for Mtr4 to the exosome^10^. In addition to providing RNA unwinding activity, Mtr4 also forms various adaptor complexes to deliver substrates to the nuclear exosome. The best-characterized adaptor complex is the TRAMP (Trf4/Air2/Mtr4 polyadenylation) complex, which is involved in nuclear surveillance and turnover of sn/snoRNAs, pre-rRNAs, mRNAs, and ncRNAs. In *Saccharomyces cerevisiae* (*S. cerevisiae*), the TRAMP complex is composed of Mtr4p, noncanonical poly(A) polymerase Trf4p/Trf5p, and zinc-knuckle protein Air1p/Air2p^11^. In yeast, it has been shown that TRAMP facilitates degradation of hypomodified initiator tRNA^Met^^12^ and cryptic unstable transcripts (CUTs) produced by Pol II^13^.

In humans, Mtr4 (hMTR4) participates in several other complexes. One of them is the nuclear exosome-targeting (NEXT) complex, which contains the scaffolding zinc-knuckle protein ZCCHC8 and the RNA-binding protein RBM7 (refs. ^14–17^). Substrates of NEXT are primarily early and unprocessed unadenylated (pA-) RNAs, promoter upstream transcripts (PROMPTs), and enhancer RNAs (eRNAs)^17,18^. More recent work described the zinc-finger protein ZFC3H1, which bridges hMTR4 with the nuclear poly(A)-binding protein PABPN1, termed the pA-tail-exosome-targeting (PAXT) connection^19^. PAXT is mainly responsible for the degradation of polyadenylated RNAs and snoRNA host gene transcripts^19^. Interestingly, hMTR4 takes part in the formation of NEXT and PAXT in a mutually exclusive manner^19^.

Studies performed in fission yeast *Schizosaccharomyces pombe* (*S. pombe*) led to the discovery of a Mtr4 paralogue, Mtl1 (52% identity and 73% similarity over 90% of the sequence)^20^. Mtl1 interacts with the zinc-finger protein Red1 (ZFC3H1 in human), forming the core of the 11-subunit MTREC (*Mt*l1–*Re*d1 *c*ore) complex^20,21^, also known as NURS (nuclear RNA silencing) complex^22^. The MTREC complex is functionally equivalent to the human NEXT and PAXT complexes, while the composition of its subunits is very closely related to the PAXT complex (orthologous gene pairs between human PAXT- and *S. pombe* MTREC complexes: hMTR4–*sp*Mtl1; ZFC3H1–*sp*Red1; PABPN1–*sp*Pab2). In addition, the CBCA submodule of the MTREC complex (*sp*Cbc1, *sp*Cbc2, and *sp*Ars2 subunits) is the direct orthologue of the human CBCA complex that interacts with both the human NEXT and PAXT complexes possibly through the zinc-finger protein ZC3H18 (known also as NHN1)^19^. Thus, the biochemical and functional characterization of the MTREC complex provides valuable insights into the functional and mechanistic understanding of the human PAXT and NEXT complexes. The MTREC complex also has additional subunits with close human homologues: (i) the zinc-finger protein *sp*Red5—closest human homologue is ZC3H3^23^; (ii) the RNA-binding protein *sp*Rmn1—closest homologues are RBM26/RBM27 (ref. ^23^); (iii) the canonical poly(A) polymerase *sp*Pla1—orthologue of human PAPOLA/G/B; and (iv) the YTH-family RNA-binding protein *sp*Mmi1—closest homologues are YTHDF1/2/3. It remains to be seen if these subunits are specific to *S. pombe* or whether they represent subunits of the human NEXT/PAXT complexes or other yet unidentified human exosome targeting complexes.

Here, we analyze the submodule organization of the MTREC complex using yeast two-hybrid (Y2H) and pull-down assays, and identify the direct interaction partners of Red1. The zinc-finger protein Red1 serves as the main scaffold for the entire MTREC complex, assembling the individual submodules, including the Mtl1 helicase, into a large complex. We determined the regions required for the interaction between Red1 and individual MTREC subunits including the poly(A) polymerase, Pla1. We reconstituted and solved the crystal structure of the minimal Mtl1–Red1 complex at 1.99 Å resolution to gain mechanistic insights into the MTREC core. We determined the residues that are crucial for the interaction and further validated them by mutational analysis in vitro and in vivo. Surprisingly, abolishing the interaction between Mtl1 and Red1, using either Red1 or Mtl1 point mutants, is lethal for the cells, highlighting the importance of a functional interaction between the Mtl1 helicase and the rest of the MTREC complex. Notably, in our structure Red1 binds to both the KOW domain and stalk helices of the helicase. This interaction extends the canonical interface previously seen in other helicase-adaptor complexes.

## Results

### Red1 serves as scaffold for MTREC assembly

**Fig. 1:**
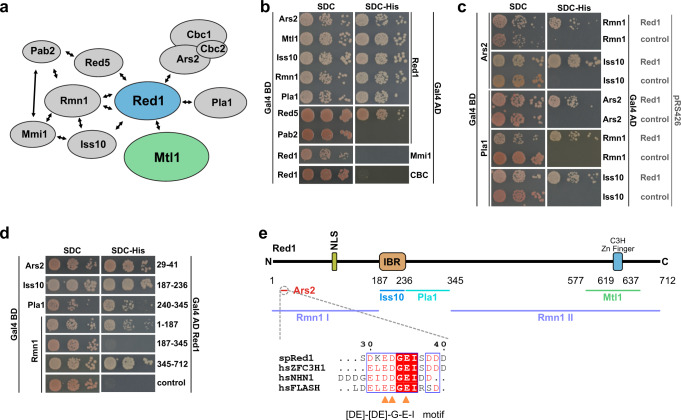
Detailed analysis of MTREC submodule interactions. **a** Organization of the MTREC complex. The Mtl1–Red1 core interacts via Red1 with all submodules, comprising Cbc1–Cbc2–Ars2, Iss10–Mmi1, Red5–Pab2–Rmn1, and Pla1. Arrows indicate all direct interactions identified in this study (see **b** and **c**). **b** Yeast two-hybrid analysis identified direct interactions between Red1 and Ars2, Mtl1, Iss10, Rmn1, Pla1, and Red5. For the CBC complex, Cbc1 is fused to Gal4 AD and an untagged version of Cbc2 is expressed from a third plasmid, pRS426. Gal4 BD, Gal4 DNA-binding domain; Gal4 AD, Gal4 activation domain; SDC-Leu-Trp (SDC) and SDC-Leu-Trp-His (SDC-His). Auto-activation controls are shown in Supplementary Fig. 1a. **c** Yeast three-hybrid analysis shows that interactions between Ars2-Rmn1, Ars2-Iss10, Pla1-Ars2, Pla1-Rmn1, and Pla1-Iss10 are bridged by Red1. SDC-Leu-Trp-Ura (SDC) and SDC-Leu-Trp-Ura-His (SDC-His). **d** Yeast two-hybrid analysis shows that the different submodules interact with various Red1 truncation variants. **e** Scheme of the interacting regions of the MTREC submodules with Red1 analyzed in **d**. The inset shows a multiple sequence alignment of the Ars2-binding region present in *sp*Red1, *hs*ZFC3H1, *hs*NHN1, and *hs*FLASH. IBR Iss10-binding region, NLS nuclear localization sequence.

Purifications of the MTREC complex from *S. pombe* cells suggest that it is composed of a Mtl1–Red1 core module and four submodules: Cbc1–Cbc2–Ars2 (CBCA), Red5–Pab2–Rmn1, Iss10–Mmi1, and Pla1 (refs. ^21,22^). To further dissect the organization of this large, 11-subunit complex, we used Y2H analysis to identify direct and indirect interactions within the complex. Our Y2H results revealed that the Red1 subunit forms the main scaffold of the entire MTREC complex, connecting the Mtl1 helicase with the individual submodules (Fig. 1a, b and Supplementary Fig. 1a, b). As interactions identified with Y2H can also be mediated by protein–RNA–protein interaction, we have taken into consideration the immunoprecipitation results of the different MTREC submodules performed in the presence of benzonase previously^21^. In addition to the Red1–Mtl1 interaction, we found that Red1 also binds directly to Ars2, Rmn1, Red5, Iss10, and the poly(A) polymerase, Pla1.

Next, we asked if Red1 could bind two different submodules simultaneously. To answer this question, we performed Y3H assays, where a positive readout is observed only when an interaction between the three components occurs. As an example, in the presence of Red1, expressed from a third plasmid, Ars2 and Rmn1 show an interaction in the Y3H system (Fig. 1c). However, interaction is not observed between Ars2 and Rmn1 when a control plasmid is used instead of the Red1-expressing plasmid (no growth on SDC-His media). Likewise, interaction between the non-interacting pairs Ars2-Iss10, Pla1-Ars2, Pla1-Rmn1, and Pla1-Iss10 is mediated with a third plasmid expressing Red1, while an empty vector could not restore growth on SDC-His (Fig. 1c). Furthermore, Ars2 bridges the interaction between Red1 and Cbc1 (CBC), forming the CBCA (Cbc1–Cbc2–Ars2) submodule, whereas Rmn1 and Iss10 bridge the interaction of Red1 with Pab2 and Mmi1, respectively (Supplementary Fig. 1c). These results strongly suggest that Red1 acts as a scaffold for the formation of the MTREC complex and the individual submodules can bind simultaneously to this scaffold.

Interestingly, we could not identify direct interactions between Mtl1 and MTREC submodules (Supplementary Fig. 1b), suggesting that Red1 might be solely responsible for connecting the Mtl1 helicase to the rest of the MTREC complex. In Fig. 1a we have summarized all interactions within the MTREC complex that were demonstrated in our Y2H experiments.

### MTREC submodules use independent binding sites on Red1

To determine the Red1 regions responsible for the interactions with individual submodules, we used various Red1 N- and C-terminal deletion constructs. This analysis revealed that each submodule has a dedicated, non-overlapping binding site within the Red1 scaffold (Fig. 1d, e). The interacting region for Ars2 localizes at the Red1 N-terminus (residues 29–41), for Iss10 at Red1 residues 187–236, and for Pla1 at Red1 residues 240–345 (Fig. 1d, e and Supplementary Fig. 1d). Interestingly, Rmn1 interacts with two distinct regions within Red1, as both Red1 N-terminal (residues 1–187) and C-terminal regions (residues 345–712) can strongly bind Rmn1 (Fig. 1d, e). We were able to narrow down the Red1-binding site within Ars2 to a region close to the Ars2 C-terminus (residues 450–516) (Supplementary Fig. 1e, right panel). Since a human FLASH peptide was shown to interact within the corresponding region of human ARS2 (ref. ^24^), we analyzed all known Ars2-binding partners for common motifs that might bind to this region. Indeed, we were able to identify a short, evolutionarily conserved binding motif of [DE]–[DE]–G–E–I within *sp*Red1 and also within human NHN1 (ZC3H18) and FLASH proteins, the known interaction partners of human ARS2 (Fig. 1e). Interestingly, ZFC3H1, the human orthologue of *sp*Red1 also contains the Ars2-binding motif, although it is currently thought to only interact indirectly with ARS2 via ZC3H18 (ref. ^19^). The presence of this motif within human ZFC3H1 suggests that similar to the MTREC complex, the human PAXT complex might also interact directly with the CBCA complex.

In vitro-binding experiments using full-length *sp*Ars2 and a GST-tagged version of the *sp*Red1 peptide (SDKEDGEISEDDP, containing the identified binding motif) confirmed the observed interaction. Replacing *sp*Red1 D33 and E35 by alanine residues (D33A, E35A) fully obliterated binding in our in vitro assay (Supplementary Fig. 1f); however, full-length *sp*Red1_(D33A, E35A)_ retained some residual interaction with *sp*Ars2 in Y2H experiments (Supplementary Fig. 1e). We introduced a third mutation, E32A, and our Y2H experiments confirmed that the triple mutant of Red1_(E32A, D33A, E35A)_ shows no residual binding to full-length *sp*Ars2 (Supplementary Fig. 1e). Taken together, our data show that the different submodules can associate with Red1 simultaneously, and that the CBC complex is recruited via Ars2.

### Analysis of Red1 interaction with Red5–Pab2–Rmn1 and Mmi–Iss10 submodules

**Fig. 2:**
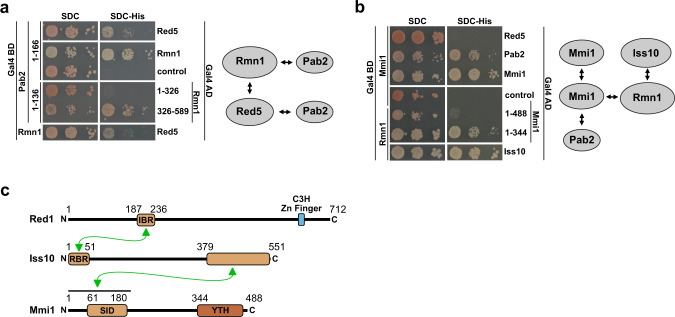
Interaction analysis of Red5–Pab2–Rmn1 and Mmi–Iss10 submodules. **a** Y2H analysis identified interactions within the Red5–Pab2–Rmn1 submodule. **b** Y2H analysis identified interactions between the Iss10–Mmi1 and Red5–Pab2–Rmn1 submodules. **c** Scheme summarizing the interactions of Red1, Iss10, and Mmi1. The minimal interacting regions identified in this study are depicted. IBR Iss10-binding region, RBR Red1-binding region, SID self-interaction domain, Gal4 BD Gal4 DNA-binding domain, Gal4 AD Gal4 activation domain, SDC-Leu-Trp (SDC) and SDC-Leu-Trp-His (SDC-His).

Using Y2H analysis, we found that Pab2 interacts directly with both Rmn1 and Red5 (Fig. 2a). The Pab2 C-terminal region (residues 137–166) is dispensable for the interaction with the Rmn1 C-terminal region (residues 326–589) (Fig. 2a). We confirmed the previously identified Red1–Iss10 (ref. ^25^) and Iss10–Mmi1 interactions involved in Iss10–Mmi1 submodule formation (Supplementary Fig. 2a), as well as the ability of Mmi1 to self-interact^26^ (Fig. 2b). Our Y2H assays also revealed a tight interaction between the Iss10–Mmi1 submodule and the Red5–Pab2–Rmn1 submodule through a direct interaction between Mmi1–Rmn1, Mmi1–Pab2, and Iss10–Rmn1 (Fig. 2b), suggesting that these five proteins might form one functional submodule within the MTREC complex. We also narrowed down the Red1 interaction site within Iss10 to the N-terminus (residues 1–51) (Fig. 2c and Supplementary Fig. 2b), and we could confirm the Red1–Iss10 interaction by in vitro GST pull-down assays using Red1_187–236_ and Iss10_1–51_ fragments (Supplementary Fig. 2c). The in vitro-reconstituted Red1_187–236_–Iss10_1-51_ complex was stable even after washing with high salt (1.5 M NaCl). We validated the previously reported Iss10–Mmi1 interaction^25^ in our Y2H studies, and localized the interaction to the Iss10 the C-terminal region (residues 379–551) and to the Mmi1 N-terminal domain (residues 1–191, Fig. 2c and Supplementary Fig. 2a).

### The Mtl1 arch binds adjacent to the Red1 zinc-finger domain

**Fig. 3:**
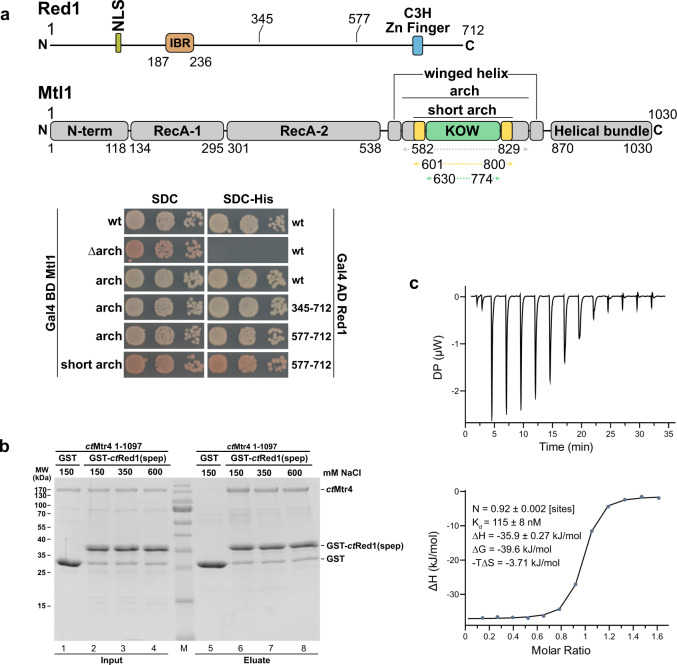
Characterization of the Mtl1–Red1 core complex. **a** Domain organization of Red1 and Mtl1. The arch construct contains the full stalk and the KOW domain (green), whereas the short arch contains a shortened stalk (missing the first and the last helices, yellow) and the KOW domain (top panel). The relevant boundaries of the constructs used in the current study are shown with residue numbers for both proteins. Yeast two-hybrid analysis of Red1 and Mtl1 shows an interaction of the Mtl1 short arch and the Red1 C-terminal region (lower panel). A scheme with all constructs used here is provided in Supplementary Fig. 2a. **b** Coomassie stained SDS-PAGE of an in vitro-binding assay of Mtr4 and Red1 from *Chaetomium thermophilum* (*ct*). *ct*Mtr4 (full-length) interacts with GST-*ct*Red1_1040–1091_. This interaction is stable under high salt conditions (600 mM NaCl). **c** ITC measurement of *ct*Red1_1040–1091_ (cell) and *ct*Mtr4_SA_ (syringe). *K*_D_ and thermodynamic parameters are shown. The ITC measurement was performed in duplicate.

As the RNA helicase is important for MTREC activity, we set out to further characterize the Mtl1–Red1 interaction. Mtl1 has a similar domain organization as the well-characterized yeast RNA helicase Mtr4 (ref. ^27^), and consists of an N-terminal low-complexity region, a helicase domain with an arch insertion (comprising a stalk and KOW domain) and a C-terminal helical bundle (Fig. 3a and Supplementary Fig. 3a). In contrast, Red1 has no predicted, folded domains besides a zinc-finger domain close to the C-terminus (residues 618–639). To dissect the Mtl1–Red1 interaction we performed Y2H and GST pull-down experiments. While full-length Mtl1 interacts with Red1, the Mtl1_∆arch_ (Δ582–829) variant does not, suggesting that binding requires the arch (Fig. 3a, lower panel). Based on these data, we created Mtl1 truncation variants containing either the arch domain only (Mtl1_A_, residues 582–829) or a shorter version of the arch domain (Mtl1_SA_, residues 601–800). Indeed, both variants interact with Red1 similarly to the full-length Mtl1 (Fig. 3a, lower panel). Next, we generated an Mtl1 variant lacking the KOW domain (Mtl1_∆KOW_, Δ630–774), which shows severely decreased binding (Supplementary Fig. 3b, no growth on SDC-Ade). These data suggest that Red1 binds to a shared surface between the Mtl1 stalk and the KOW domain. We were also able to narrow down the binding region within the Red1 protein to residues 345–712 (Fig. 3a). Further removal of the N-terminal 576 residues (residues 577–712) still allowed interaction with Mtl1_A_. This truncation also showed strong interaction with the Mtl1_SA_ (Supplementary Fig. 3b, c). Interestingly, this short Red1 region (residues 577–712) contains a predicted zinc-finger domain with a CX_8_CX_4_C_3_H motif, which was previously shown to have RNA-binding activity^28^. Using multiple sequence alignments of Red1 homologues, we identified a conserved region in *sp*Red1, spanning residues 577–653 (Supplementary Fig. 4). We hypothesized that this short and conserved region might be responsible for the interaction with Mtl1 and decided to test this in vitro. Efforts to recombinantly express and purify the Mtl1–Red1 complex from *S. pombe* were unsuccessful. Therefore, we switched to the *Chaetomium thermophilum* (*ct*) Mtr4–Red1 complex (homologues of *sp*Mtl1/*sp*Mtr4 and *sp*Red1) due to superior biochemical properties of proteins and complexes from this thermophilic eukaryote^29^. Bioinformatic analysis and sequence alignments confirmed that the MTREC complex is conserved in *C. thermophilum* (Supplementary Figs. 5–7). Initial trials to recombinantly purify the corresponding *ct*Red1 peptide (*ct*Red1_pep_, residues 1014–1091) alone were not successful; therefore, we generated a shorter peptide comprising residues 1040–1091 (*ct*Red1_spep_). Using a GST pull-down experiment, we observed strong binding between GST-*ct*Red1_spep_ and full-length *ct*Mtr4, which confirms that this region is sufficient for binding not only in *S. pombe* but also in *C. thermophilum* (Fig. 3b). This interaction tolerated high salt (up to 600 mM NaCl), suggesting a hydrophobic interface. To biochemically characterize this interaction, we determined the affinity between *ct*Red1_spep_ and *ct*Mtr4_SA_ (residues 654–865) using isothermal titration calorimetry (ITC). We obtained a 1:1 binding stoichiometry and nanomolar affinity (115 ± 8 nM, Fig. 3c). Taken together, our in vitro-binding assays confirmed that the C-terminal region of *ct*Red1, containing the zinc-finger domain, can bind to the short arch region of *ct*Mtr4 with high affinity.

### Crystal structure of the Mtr4–Red1 complex

**Fig. 4:**
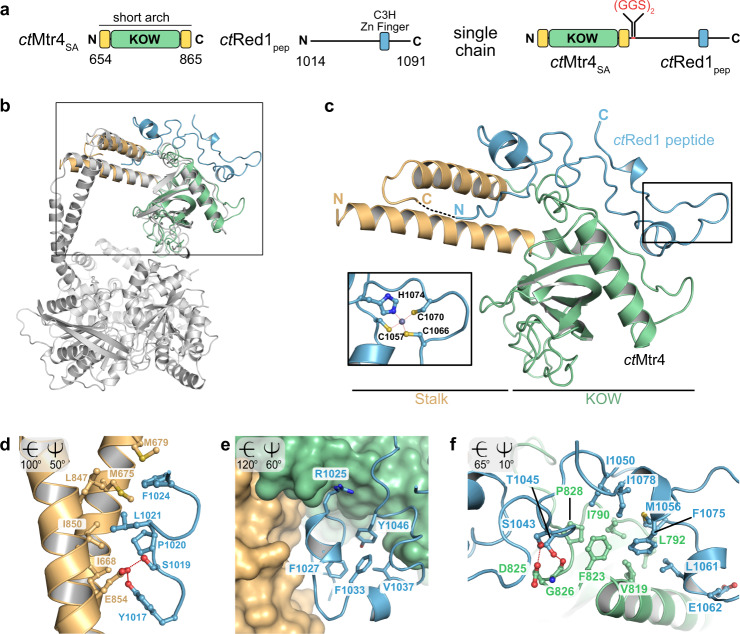
Crystal structure of *ct*Mtr4–*ct*Red1 and interface characterization. **a** Domain organization of the crystallization constructs of *ct*Mtr4 and *ct*Red1 are shown. The *ct*Mtr4_SA_ and *ct*Red1_pep_ are linked with a GS (glycine–serine) linker to form the single-chain construct. **b** Crystal structure of the *ct*Mtr4_SA_–*ct*Red1_pep_ complex superimposed on the structure of the full-length Mtr4 from *Saccharomyces cerevisiae* (gray*;* PDB: 2XGJ^27^). The stalk (light-orange) and KOW (green) domain are structurally highly similar. **c** Overview of the *ct*Mtr4_SA_–*ct*Red1_pep_ complex. The *ct*Red1 peptide (light blue) interacts with the stalk and the KOW domain of *ct*Mtr4. The *ct*Red1 Zn-finger is located close to the C-terminus (inset). The Cys- and His-residues required for Zn-finger formation are conserved. **d** *ct*Red1_pep_ N-terminal region interacts with the stalk helices through hydrogen bonds and van der Waals contacts. **e** Red1 forms a U-shaped structure stabilized by hydrophobic residues, which inserts between the stalk helices and the KOW domain. **f** Multiple hydrophobic residues form a tight interaction between *ct*Mtr4 KOW domain and *ct*Red1_pep_. Rotation angles relative to the view in **c** are shown at the top left corner of **d**–**f**.

In order to obtain structural insights into the *ct*Mtr4–*ct*Red1 complex, we performed crystallization trials using full-length *ct*Mtr4 and several *ct*Red1 peptides. Although crystals were obtained in various conditions and the structures were readily determined, we did not observe electron density for the KOW domain and/or the *ct*Red1 peptides. We therefore decided to explore different strategies, including generating a single-chain construct in which *ct*Red1_pep_ is fused to the C-terminus of *ct*Mtr4_SA_ via a short linker (*ct*Mtr4_SA_–*ct*Red1_pep_ construct). This strategy has been successfully employed previously^30^ (Fig. 4a). The single-chain *ct*Mtr4_SA_–*ct*Red1_pep_ complex was readily crystallized and the crystals diffracted to 1.99 Å resolution. The structure was determined de novo using the anomalous signal of the bound Zn^2+^ (Zn-SAD), whose identity was confirmed by X-ray fluorescence (XRF) emission spectrum analysis (Supplementary Fig. 8a). Initial electron density was easily interpretable and allowed for automated/manual model building (Supplementary Fig. 8b). Data collection and refinement statistics are summarized in Table 1. The asymmetric unit (ASU) contains two molecules of the single-chain complex, which exhibit very small differences as indicated by the low root-mean-square deviation (RMSD) of 1.02 Å for Cα atoms of 269 residues. The orientation of the stalk helices with respect to the KOW domain are different between both complexes, indicating flexibility. Although the ASU contains a crystallographic dimer (Supplementary Fig. 9a), dimerization could not be observed in solution as analyzed by multi-angle light scattering (SEC-MALS) (Supplementary Fig. 9b, c). The *ct*Mtr4 short arch superimposed well with the *sc*Mtr4 structure^27^, revealing that the *ct*Red1 peptide is positioned on top of the KOW domain (Fig. 4b, c). Overall, the *ct*Red1 peptide can be divided into two regions. The N-terminal region (residues 1017–1024) of the peptide contains several highly conserved residues, among them Tyr1017 and Ser1019, which form hydrogen bonds with Glu854 from the stalk (Fig. 4d). Furthermore, Pro1020, Leu1021, and Phe1024 of *ct*Red1_pep_ pack against Ile668, Met675, Met679, Leu847, and Ile850 of the stalk helices (Fig. 4d). These interactions are followed by a U-shaped structure (residues 1025–1046), which is positioned between the stalk-interacting and the KOW-interacting residues of the peptide (Fig. 4e). The U-shaped structure is stabilized by a hydrophobic cluster comprising Phe1027, Phe1033, Val1037, and Tyr1046, which potentially impart rigidity to the peptide. Residues 1047–1080 exclusively interact with the KOW domain, including more hydrophobic interactions mediated by Ile1050, Met1056, Leu1061, Phe1075, and Ile1078, which pack against Ile790, Leu792, Val819, Phe823, and Pro828 (Fig. 4f). Also, *ct*Red1Ser1043 and Thr1045 form hydrogen bonds with *ct*Mtr4Asp825 and Gly826, respectively. The middle part of the peptide (residues 1047–1056) is stabilized and held in position by the conserved Zn-finger of *ct*Red1, composed of Cys1057, Cys1066, Cys1070, and His1074 (Fig. 4c, inset). Overall, the interaction between *ct*Mtr4_SA_ and *ct*Red1_pep_ covers 1319.8 and 1403.2 Å^2^ on their respective solvent accessible surface, which corresponds to 10.3 and 26.7% of the total solvent accessible surface, respectively (Supplementary Fig. 10a). A detailed analysis using PISA web server^31^ of the residues involved in the interaction is shown in Supplementary Fig. 11. The majority of residues within this interface is conserved (Supplementary Fig. 10b). With these data at hand, we also succeeded in determining the structure of a complex comprising co-expressed (but not fused; split chain complex) *ct*Mtr4_SA_ and *ct*Red1_pep_, albeit at a lower resolution of 2.75 Å. Comparison of this structure with the single-chain structure revealed only small differences, as indicated by the low RMSD of 1.08 Å for Cα atoms of 273 residues (Supplementary Fig. 10c), thus validating the single-chain structure. The extended loops connecting β-strand β2–β3 (loop1) and β3–β4 (loop2) of the KOW domain do not participate in the interaction and their deletion does not interfere with Red1 binding (Supplementary Fig. 12).

**Table 1:** Data collection and refinement statistics.

	*ct*Mtr4_SA_/*ct*Red1 single-chain	*ct*Mtr4_SA_/*ct*Red1 split chain
**Data collection**		
Beamline	ESRF ID29	ESRF ID29
Wavelength (Å)	1.27411	1.27411
Space group	*P*2_1_2_1_2_1_	*P*6_5_22
Cell dimensions		
* a*, *b*, *c* (Å)	44.98, 88.90, 168.36	172.19, 172.18, 145.08
* α*, *β*, *γ* (°)	90, 90, 90	90, 90, 120
Resolution (Å)	47.46–1.99 (2.061–1.99)^a^	49.71–2.75 (2.848–2.75)^a^
*R*_merge_	0.1033 (1.332)	0.129 (3.048)
*I*/*σI*	13.45 (1.55)	25.61 (1.52)
Reflections total	614,526 (57,745)	1,307,677 (122,975)
Reflections unique	47,338 (4660)	33,467 (3283)
Completeness (%)	99.91 (99.79)	99.95 (100.00)
Multiplicity	13.0 (12.4)	39.1 (37.5)
**Refinement**		
*R*_work_	0.2119 (0.3222)	0.2174 (0.3432)
*R*_free_	0.2470 (0.3506)	0.2573 (0.3720)
No. atoms		
Protein	4489	4582
Water	180	0
Ligands	42	17
B-factors		
Protein	54.96	105.71
Water	52.27	–
Ligands	67.93	137.33
R.m.s deviations		
Bond lengths (Å)	0.006	0.010
Bond angles (°)	1.09	1.35
Ramachandran plot		
Favored (%)	98.72	95.89
Allowed (%)	1.28	3.58
Outliers (%)	0.00	0.54

^a^Values in parenthesis refer to the highest resolution shell.

### Identification of critical interface residues

**Fig. 5:**
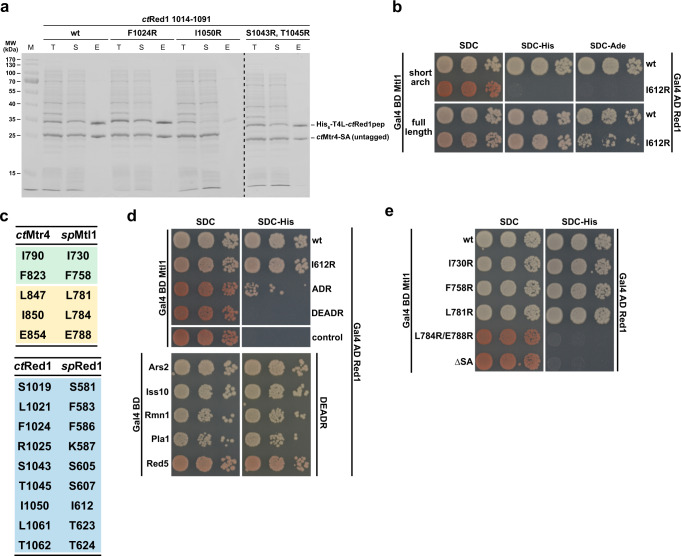
Mutation analysis of the Mtr4/Mtl1–Red1 interface. **a** Coomassie stained SDS-PAGE analysis of co-expression and purification of T4L-*ct*Red1_pep_ wild type (wt) and mutant variants. While wt T4L-*ct*Red1_pep_ co-purifies untagged *ct*Mtr4_SA_, the I1050R mutant does not. F1024R and the double S1043R, T1045R mutants had no effect on the *ct*Mtr4–*ct*Red1 interaction. T Total, S soluble, E eluate. **b** Y2H analysis of *sp*Red1 I612R (I1050R in *ct*Red1) and *sp*Mtl1 short arch or full-length variants. While the short arch shows no binding, the full-length *sp*Mtl1 retained binding. **c** Table showing the mutated residues in Mtl1/Red1 and Mtr4/Red1 in *S. pombe* (*sp*) and *C. thermophilum* (*ct*), respectively (color code is as in Fig. 4). **d** The *sp*Red1 I612R mutant binds like wt. Mutations at I612R, F586A, and K587D (ADR) weakened the interaction. Addition of S581D and F583E mutations (DEADR variant) completely abolished binding. The DEADR variant retained binding to the remaining interaction partners in MTREC, which confirmed proper protein expression. **e** Y2H analysis of *sp*Mtl1 mutants for *sp*Red1 binding. The single-residue I730R, F758R, and L781R mutants showed no reduction in binding. The L784R, E788R double mutant did not interact with *sp*Red1, similar to our observation for the short arch deletion (∆SA).

After analyzing the *ct*Mtr4_SA_–*ct*Red1_pep_ interface in the crystal structure, we set out to disrupt the complex by introducing mutations in *ct*Red1 and also *sp*Red1. We identified four residues that might be critical for the interaction: Phe1024, Ser1043, Thr1045, and Ile1050. Using a co-expression strategy of His-tagged *ct*Red1_pep_ and untagged *ct*Mtr4_SA,_ we analyzed the effect of the different mutants on complex formation. Notably, when *ct*Red1_pep_ fails to interact with *ct*Mtr4_SA_ it becomes insoluble and is therefore not visible in the soluble or elution fractions (Fig. 5a). To our surprise, *ct*Red1_pep_ harboring either the F1024R or S1043R/T1045R mutations was still able to interact with *ct*Mtr4_SA_ in vitro. However, replacing Ile1050 by an arginine (I1050R) completely abolished the interaction (Fig. 5a). We mutated the corresponding residue in full-length *sp*Red1 to arginine (I612R) and analyzed binding to *sp*Mtl1 in our Y2H assay. Recapitulating the in vitro data, the *sp*Red1(I612R) mutant did not interact with the *sp*Mtl1_SA_ construct; however, full-length *sp*Mtl1 retained its interaction with *sp*Red1(I612R) (Fig. 5b). These data also underline the high conservation of these MTREC components and their interaction between *C. thermophilum* and *S. pombe*. In addition, two more residues were identified in our structure as critical for helicase binding, and therefore were changed in *sp*Red1 (see Fig. 5c for corresponding residues in *C. thermophilum* and *S. pombe*). As the *ct*Red1–*ct*Mtr4 complex has a large interface, which to a significant extent involves main chain interactions, we mainly introduced reverse charge mutations and replaced small residues with big bulky residues (e.g. arginine) in order to abolish the interaction. The resulting triple mutant F586A, K587D, and I612R (ADR) showed a strongly impaired interaction with *sp*Mtl1; however, binding was not completely eliminated. Next, we introduced two additional mutations in the *sp*Red1 ADR mutant (S581D and F583E, DEADR) to weaken the interaction with the stalk domain of *sp*Mtl1. This variant completely abolished binding to full-length *sp*Mtl1 (Fig. 5d). To confirm that the overall stability of the DEADR variant is not affected in the Y2H system, we analyzed its interaction with the remaining MTREC subunits (Fig. 5d). The interaction strength of the DEADR variant is similar to wild-type *sp*Red1.

We also introduced mutations in *sp*Mtl1 to eliminate its interaction with *sp*Red1 and assessed the interaction by Y2H assays. The three single-residue mutants I730R, F758R, and L781R interacted with *sp*Red1 similarly to the wild type. Combining L784R and E788R, which are both located in the Mtl1 stalk domain, completely abolished the interaction with Red1. Similarly, when we deleted the *sp*Mtl1 short arch the interaction was completely lost (Fig. 5e). Interestingly, the residues that are most critical for the Red1–Mtl1 interaction are located upstream of the Red1 Zn-finger domain. They interact specifically with the Mtl1 stalk domain as seen in our structure, suggesting that the stalk helices are central to the interaction. Overall, Red1 establishes an extended binding interface with Mtl1 that has not been observed before.

### The interaction between Mtl1 and Red1 is critical for cell survival

**Fig. 6:**
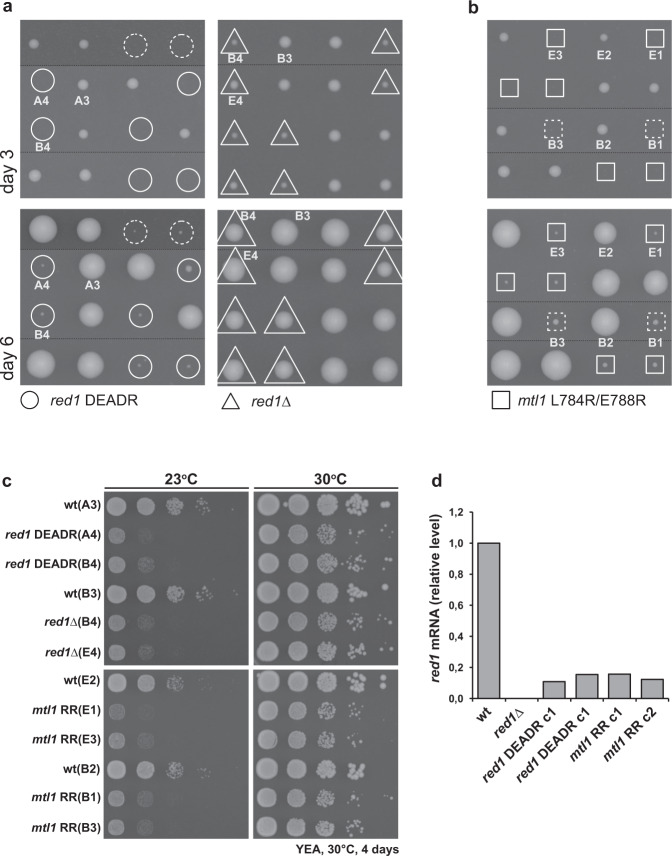
In vivo analysis of *red1 DEADR* and *mtl1 RR* variants. **a** Tetrad dissection of *red1 DEADR* variant and *red1*∆. The wild type (wt) colonies grew after 3 days, whereas the *DEADR* variant (circles) appeared after 6 days. *red1*∆ colonies (triangles) grew similarly to wt colonies. **b** Tetrad dissection of *mtl1* L784R/E788R (*RR*) variant. The wt colonies grew after 3 days, whereas *mtl1 RR* (squares) appeared after 6 days. **c** Phenotypic growth assay after 4 days at different temperatures. The *red1 DEADR* variant, *red1*∆, *mtl1 RR,* and respective wt, were spotted after serial dilutions. The growth of the mutant strains at 30 °C is comparable to wt; however, at 23 °C all mutants exhibit a slow growth phenotype. **d** qPCR analysis of the *red1* mRNA expression level. For the analysis, *red1 DEADR* (dotted circles) and *mtl1 RR* (dotted squares) were used together with wild type and *red1*∆ strains. Two biological replicates were performed (labeled with c1 and c2), *n* = 2. Source data are provided as a Source Data file.

Our Y2H results demonstrate that the *sp*Red1 DEADR variant is not able to interact with *sp*Mtl1, while its interaction with other MTREC subunits is not affected. To gain further insights into the specific function of *sp*Mtl1 within the MTREC complex, without interfering with its MTREC-independent functions, we decided to test the effects of the *sp*Red1 DEADR mutant in vivo. Since our attempts to introduce the *red1*_*DEADR*_ into the haploid *S. pombe* genome have repeatedly failed, we used diploid *S. pombe* cells to generate heterozygous *red1*_*DEADR*_/*red1*_*wt*_ strains. After confirming the mutations by PCR and by sequencing the *red1* gene, we induced sporulation to generate haploid strains carrying the *red1*_*DEADR*_ allele. To our surprise, only half of the spores from the resulting tetrads were viable, and these spores exclusively carried the *red1*_*wt*_ allele (Fig. 6a), suggesting that the *red1*_*DEADR*_ allele is lethal for the cells. As a comparison, complete deletion of the *red1* gene leads to viable spores and, in agreement with previous reports^21,28^, only a slight growth defect can be observed in the *red1∆* haploid cells, compared to wt (Fig. 6c). To verify whether the lethality is caused by the dissociation of Mtl1 from the MTREC complex, we integrated our Red1 interaction-deficient *mtl1* allele (*mtl1*_*L784R/E788R*_) into diploid *S. pombe* cells and analyzed the haploid progeny after sporulation. Similar to the Mtl1 interaction-deficient *red1* allele (*red1*_*DEADR*_), cells carrying the *mtl1*_*L784R/E788R*_ allele are not viable (Fig. 6b). This further confirms the surprising observation that, while the entire MTREC complex is not essential for cell survival, a “truncated” MTREC complex missing Mtl1 is lethal for cells.

Interestingly, after an extended period of time, tiny colonies appeared both at the *red1*_*DEADR*_ spores and at the *mtl1*_*L784R/E788R*_ spores (Fig. 6a, b—day 6). However, after recovering these colonies, they regained fitness and showed only a slight growth defect, similar to *red1∆* cells (Fig. 6c). We confirmed that these cells were haploid cells carrying the *red1*_*DEADR*_ or the *mtl1*_*L784R/E788R*_ alleles. However, qPCR results revealed that the expression level of the *red1* gene was diminished to below 15% in all of the investigated colonies (two colonies of *red1*_*DEADR*_ and two colonies of *mtl1*_*L784R/E788R*_) compared to wt (Fig. 6d). These results suggest that the small colonies are escape mutants that managed to suppress their lethal *red1*_*DEADR*_ or *mtl1*_*L784RE788R*_ alleles by repressing (genetically or epigenetically) the expression level of the *red1* gene, effectively becoming a *red1-null* allele. We did not identify any genetic mutations in the *red1* gene or in the promoter region (except for the introduced mutations of the *red1*_*DEADR*_ allele in the two corresponding strains), suggesting that epigenetic mechanisms might play a role in this process. Notwithstanding, the appearance of these escape mutants further confirmed our original observation that separating the Mtl1 subunit from an otherwise undisturbed MTREC complex is incompatible with cell survival. A possible hypothesis that could explain the unexpected lethal phenotype of the *red1*_*DEADR*_ and *mtl1*_*L784RE788R*_ alleles is that the Mtl1-truncated MTREC complex, which is likely unable to offload its RNA cargo to the exosome, might trap and deplete some of the essential subunits, such as the CBCA complex or Red5 protein. However, overexpression of the *red1*_*DEADR*_ mutant protein in the presence of a wt *red1* allele did not lead to any growth defect (Supplementary Fig. 13), revealing that the *red1*_*DEADR*_ mutation is not a dominant-negative allele. This finding does not support the hypothesis that the lethality is likely caused by essential subunit trapping. Further research is required to identify the mechanisms underlying the surprising lethal phenotype of the Mtl1-truncated MTREC complex.

## Discussion

The identification and biochemical characterization of the MTREC complex to date has been mainly based on in vivo purifications and subsequent mass spectrometric determination of the interacting proteins^20–22^. While the Mtl1 and Red1 proteins were unanimously identified as the core complex (Mtl1–Red1 core), the exact subunit composition, and whether a large “super-complex” or several independent smaller complexes are formed, remained unclear. Here we determined the direct protein–protein interactions within the MTREC complex and outlined a detailed organization structure of this large, 11-subunit complex. We identified the Red1 protein as the main scaffold of the MTREC complex. Red1 integrates the subunits and submodules into a large complex. In contrast, the Mtl1 helicase is connected only to the Red1 scaffold and does not seem to play a central role in the overall assembly of the complex. Fine mapping of the interaction domains within Red1 revealed non-overlapping binding sites for the interacting MTREC subunits and submodules. Furthermore, our Y3H results demonstrated that the subunits and submodules can concurrently bind to the Red1 scaffold, further supporting the existence of a large MTREC “super-complex” that potentially integrates all subunits and submodules at the same time.

Our detailed Y2H-interaction map further supports the model of a modular structure of the MTREC complex, which was previously suggested by tandem-affinity purification of the complex from a *red1∆* strain^21^. We also show that the interaction between Red1 and the Pab2–Red5–Rmn1 submodule is facilitated by Rmn1 and Red5 but not Pab2. In addition, Pab2 and Rmn1 also interact with Mmi1 and Iss10, suggesting that Pab2, Red5, Rmn1, Iss10, and Mmi1 form one large functional module. We identified a direct interaction between the canonical poly(A) polymerase Pla1 and Red1. Within the MTREC complex Pla1 is required for the hyper-polyadenylation of CUTs and meiotic mRNAs, and has a critical role in the proper degradation of these transcripts^32^. This suggests that the mammalian PAXT complex might also interact directly with a poly(A)-polymerase to extensively polyadenylate substrate RNAs that are recognized by this complex. Although in our Y2H-interaction map, every known subunit of the MTREC complex is accounted for, we cannot exclude that additional interactions exist within the complex that we could not retrieve with the Y2H technique.

**Fig. 7:**
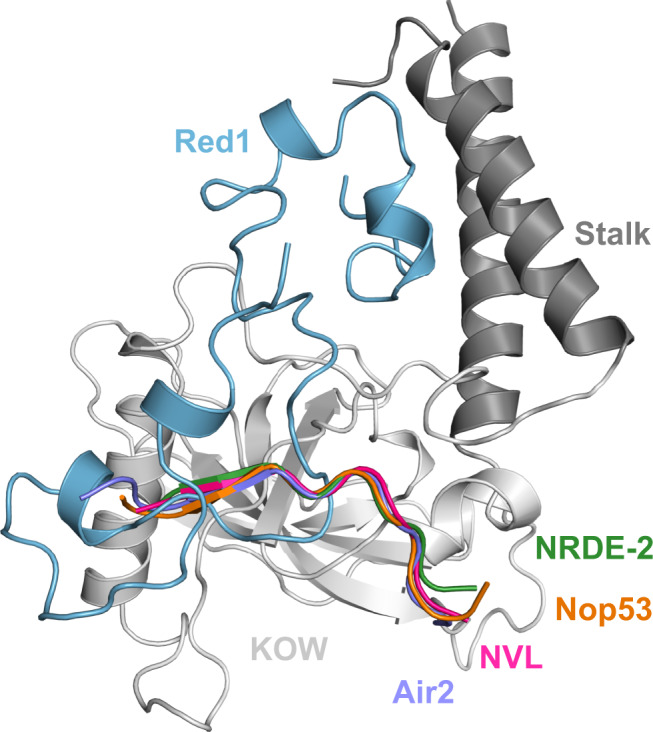
Comparison of the *ct*Mtr4–*ct*Red1 crystal structure with known Mtr4 complexes. Crystal structure of the *ct*Mtr4_SA_–*ct*Red1_pep_ complex (Red1 in blue, stalk helices in dark gray and KOW domain in light gray) is superimposed with the structures of Mtr4-Air2 (PDB: 4U4C^33^,violet), Mtr4-NVL (PDB: 6RO1 (ref. ^34^), pink), Mtr4-Nop53 (PDB: 5OOQ^35^, orange), and hMTR4-NRDE-2 (PDB: 6IEH^36^, green). For simplicity only the Mtr4 interacting peptides of these complexes are shown. A detailed comparison is shown in Supplementary Fig. 14.

The structural characterization of the Mtr4 helicase in complex with Trf4/Air2 (TRAMP)^33^, ZCCHC8 (NEXT)^15,34^, and Nop53 (ref. ^35^) (ribosome biogenesis) has provided detailed insights into the molecular interactions of Mtr4 as part of exosome adaptor complexes. So far, all of them utilize the Mtr4 KOW domain for binding distinct arch-interacting motifs (AIMs) present in, e.g., Nop53, NVL, Air2, and NRDE-2. Such detailed insights were previously not available for PAXT and MTREC complexes. In the current study, we determined a high-resolution structure of *ct*Mtr4–*ct*Red1 complex. We performed a detailed in vitro and in vivo biochemical characterization of the Mtr4–Red1 binding interface and present a conservation analysis of this binding interface in *S. pombe*. A detailed comparison of our *ct*Mtr4–*ct*Red1 structure with previously characterized structures of Mtr4 in complex with Air2 (ref. ^33^) or AIM (Nop53 (ref. ^35^), NRDE2 (ref. ^36^)) or AIM-like motif (NVL^34^) shows some similarities of the interaction interface, but also important differences (Fig. 7 and Supplementary Fig. 14). All previously characterized ligands bind to the KOW domain only and occupy a largely overlapping binding region. The N-terminal part of the AIMs interacts by β-augmentation extending the KOW domain β-sheet. While β-augmentation is not observed with Red1, a hydrophobic interface with the KOW domain is also seen with Red1. However, the conserved aspartate of the AIMs (LFXϕD) typically participates in electrostatic interactions with conserved arginine residues of the KOW domain (Supplementary Fig. 14). This interaction is not conserved in the Mtr4–Red1 complex. Importantly, in contrast to all previously characterized, canonical ligands Red1 utilizes a large interface that extends to the stalk helices and fixes them with respect to the KOW domain by inserting a U-shaped element that serves like a wedge. This extended binding interface observed for Red1 is supported by an extensive network of hydrophobic interactions and hydrogen bonds (Supplementary Fig. 14). Such a binding interface has not been reported for any other adaptor protein so far. The structure of hMTR4–NRDE2 shows that NRDE2 also forms an extended binding interface with hMTR4. However, in addition to the KOW domain it contacts the C-terminal helical bundle and the RecA domain but not the stalk helices^36^. Overall, the interaction of Mtr4 with Red1 described in our study shields the canonical adaptor protein-binding site, and suggests a mutually exclusive interaction of hMTR4 with either ZFC3H1 (the human Red1 homologue) or AIM-like motif containing adaptor proteins. Such an exclusive interaction was shown in vivo for scaffolding proteins, ZCCHC8 (AIM-like motif) and ZFC3H1 (Red1), within the NEXT and PAXT complexes, respectively^19^. Taken together, these studies show that a variety of different adaptor proteins interact with the hMTR4 KOW domain in a mutually exclusive manner, suggesting that the hMTR4 helicase can be specifically directed to different RNA-binding proteins. Interestingly, *S. pombe* has two copies of the Mtr4 helicase (Mtr4 and Mtl1) that differ in their interactions. Mtl1 is specialized for the MTREC complex through its tight interaction with Red1 (ref. ^21^), while Mtr4 is mainly involved in the formation of the TRAMP complex, but is also recruited by Utp18 and Nop53 during ribosome biogenesis^37^. At present, it is not clear why fission yeast employs two variants of the helicase. In higher eukaryotes, only one copy of the Mtr4 helicase exists that has the capacity to interact with all adaptor proteins in a mutually exclusive manner^19,34^.

The comparatively large interface between Red1 and Mtl1, which was initially hard to disrupt, suggests that this complex exists as a tight heterodimer in vivo. While Red1 deletion leads only to a moderate growth defect in *S. pombe*, mutations that break the interaction between Red1 and Mtl1 are lethal for the cells. This surprising phenotype was observed in both Mtl1 and Red1 mutants that specifically disrupt the interaction surface between the two proteins. While deletion of the Red1 protein leads to the complete disassembly of the MTREC complex and strong accumulation of CUTs and meiotic mRNAs, the Red1–Mtl1 interaction mutants result in a “truncated” MTREC complex that differs by only missing the helicase subunit. This truncated complex is likely unable to unload its RNA cargo, but it is not clear how this leads to the observed lethal phenotype.

Taken together, our data establish Red1 as the central scaffolding protein of the MTREC complex and provide the basis for future investigations addressing common principles of its human homologue. Red1 serves as a binding platform for the CBCA, Pab2–Red5–Rmn1, and Iss10–Mmi1 and the poly(A) polymerase Pla1 submodules. Therefore, Red1 essentially acts as an adaptor between Mtl1 and the various MTREC submodules to provide a route for RNA substrates to the exosome through the unwinding activity of Mtl1. The extended Mtr4–Red1 interface is crucial for viability in *S. pombe* and differs from the canonical interface previously seen in other helicase-adaptor complexes, suggesting that we might not yet have discovered the full spectrum of helicase interactions.

## Methods

### Yeast strains and genetic methods

Gene integration and C-terminal gene tagging were performed using classical yeast genetics methods. The *S. pombe* strains used in this study are listed in Supplementary Table 1.

### Plasmid constructs

The coding sequences of *ct*Mtr4 and *ct*Red1 were amplified by PCR using *C. thermophilum* cDNA as template and cloned in a modified pET24d expression plasmid, resulting in His_6_-*ct*Mtr4 and His_6_-*ct*Red1. The *ct*Red1 variant obtained from cDNA differed slightly from the Uniprot entry G0S1V1. The resulting *ct*Red1 peptides contain one additional alanine (Supplementary Fig. 15). The coding sequences of all *Schizosaccharomyces pombe* proteins were amplified by PCR and cloned in the respective vectors for Y2H experiments and expression in *E. coli*. The cloned full-length genes were used as templates to generate the truncation variants. All constructs were verified by sequencing. A full list of primers and constructs used and generated in this study are listed in Supplementary Tables 2 and 3, respectively.

### Protein expression and purification

The *ct*Mtr4_SA_–*ct*Red1_pep_ single-chain construct was expressed in *E. coli* Rosetta™ 2 (DE3) strain (Novagen) using ZYM5052 auto-induction media^38^ supplemented with kanamycin (30 µg/ml) and chloramphenicol (34 µg/ml). The cells were grown at 37 °C to an OD_600_ of 0.8–1.0, and were then shifted to 18 °C for overnight incubation (usually 16–18 h). Harvested cells were used immediately for protein purification or flash frozen in liquid nitrogen and stored at −20 °C. Lysis was performed in a buffer containing 50 mM HEPES pH 7.5, 150 mM NaCl, and 20 mM imidazole (Buffer A) using a microfluidizer (Microfluidics). The lysate was centrifuged at 50,000 × *g*, 4 °C for 30 min using a JA 25.50 rotor (Beckman-Coulter). The cleared lysate was filtered through a 0.45 µm filter and loaded on a HisTrap™ HP column (Cytiva). The column was washed with 10 column volumes (CVs) Buffer A, followed by elution with Buffer A containing 400 mM imidazole (Buffer B). The protein was further purified by size exclusion chromatography (SEC) on a HiLoad® 26/600 Superdex® 75 pg column (Cytiva) using 20 mM HEPES pH 7.5 and 150 mM NaCl (Buffer C). The purity of the protein was analyzed by sodium dodecyl sulfate polyacrylamide gel electrophoresis (SDS-PAGE) followed by Coomassie staining. For the split chain complex (“native complex”), untagged *ct*Mtr4_SA_ was co-expressed with Gb1-TEV-*ct*Red1_pep_-His_6_, by essentially the same purification procedure, however with the following modification: after elution from the HisTrap™ HP column, the complex was subjected to TEV cleavage, followed by SEC on a HiLoad® 26/600 Superdex® 75 pg column (Cytiva) using Buffer C.

### Protein crystallization, data collection, and structure determination

Protein aliquots (either freshly purified or frozen) at a concentration of 85–125 mg/ml were used for crystallization trials. Crystals of the single-chain complex were obtained with the sitting-drop vapor-diffusion method at 291 K upon mixing 2:1 volume of protein and reservoir solution (200 mM magnesium acetate and 20% PEG 3350). The split chain complex (“native complex”) crystals were obtained after mixing equal volume of protein and reservoir solution containing 100 mM MES pH 6.5 and 30% PEG 300. For data collection, crystals were cryo-protected in 20% ethylene glycol (single-chain) or 20% glycerol (split chain, “native complex”) in a reservoir solution and subsequently flash frozen in liquid nitrogen.

Diffraction data for all crystals were collected at the ESRF beamline ID29 (ref. ^39^) at the Zn-Edge (1.2741 Å wavelength) based on the XRF spectrum. Diffraction data were integrated with XDS^40^ and further processed with AIMLESS^41^ from the CCP4-package^42^. The crystal of the single-chain complex belongs to the space group *P*2_1_2_1_2_1_ with cell dimensions of *a* = 44.98 Å, *b* = 88.91 Å, *c* = 168.37 Å and *α* = *β* = *γ* = 90°, and of the native complex to the space group *P*6_5_22 with cell dimensions of *a* = 172.19 Å, *b* = 172.19 Å, *c* = 145.08 Å, and *α* = *β* = 90°, *γ* = 120°. All structures were solved by Zn-SAD with the SHELXC/D/E programs^43^ navigated with HKL2MAP^44^. Clear solutions were obtained for all datasets due to the excellent anomalous signal and partially also due to the high solvent content (“native complex”). The resulting maps were easily interpretable and therefore used for automated model building at the early steps using PHENIX^45^. Automated model building was followed by manual model building in Coot^46^ and refinement, which was performed either in REFMAC5 (ref. ^47^) or PHENIX^48^. Both structures contain two molecules in the asymmetric unit. Data collection and refinement statistics are summarized in Table 1.

### SEC-MALS

To determine the molecular weight of the purified complexes in solution, they were analyzed by SEC coupled to online MALS, using a buffer containing 20 mM HEPES pH 7.5, 150 mM NaCl. Samples were prepared using a Superdex® 75 10/300 GL column (Cytiva) connected to an ÄKTA Purifier system (Cytiva) and DAWN HELEOS Light scattering detector (Wyatt Technology). Protein concentration was determined with an online Optilab-tREX refractometer (Wyatt Technology), and data analysis was performed using the ASTRA 6.1 software.

### In vitro pull-down assays

GST pull-down assays were performed in 20 mM HEPES pH 7.5, 150 mM NaCl (or higher concentration, see description in the figure legends), 5% glycerol, and 0.1% NP-40. The bait protein was incubated with a 1.5 molar excess of prey protein in 500 µl buffer at 4 °C for 45 min. Glutathione agarose beads (Cytiva) were added and the samples were incubated for an additional 45 min at 4 °C. The wash was performed on 1 ml mobicol columns (MoBiTec) using buffer with the indicated salt concentration. Beads were washed twice with 500 µl buffer. Samples were eluted with 1× Laemmli SDS buffer and analyzed by SDS-PAGE followed by Coomassie staining.

For in vitro-binding experiments with the *ct*Red1 mutants, the proteins were co-expressed in *E. coli* Rosetta 2™ (DE3) strain (Novagen). Wild type and mutant variants of T4L-*ct*Red1_pep_-His_6_ were co-expressed with an untagged version of *ct*Mtr4_SA_. Lysis was performed in a buffer containing 50 mM HEPES pH 7.5, 150 mM NaCl and 20 mM imidazole, followed by Ni-NTA purification. Samples from total, soluble, and elution fractions were analyzed by SDS-PAGE, followed by Coomassie staining.

### Isothermal titration calorimetry (ITC)

The proteins used for the ITC measurements were extensively dialyzed against 20 mM HEPES pH 7.5 and 150 mM NaCl buffer (ITC buffer). Experiments were performed with a MicroCal PEAQ-ITC (Malvern Instruments) at 25 °C and constant stirring (750 r.p.m.). Protein concentrations were 30 µM in the cell (ZZ-*ct*Red1_spep_) and 250 µM in the syringe (*ct*Mtr4_SA_). Titration was performed with one injection of 0.4 µl followed by 12 injections of 3.0 µl of the titrant protein into the cell. The data were analyzed with MicroCal PEAQ-ITC analysis software. All ITC measurements were performed in duplicates.

### Y2H and three-hybrid assays

The full-length sequences of the proteins were cloned in high copy plasmids—pGBKT7 or pGADT7 (Clontech) or low copy plasmids—pG4BDN22 and pG4ADHAN111. The interaction pairs were analyzed after co-transformation into the PJ69-4A Y2H strain^49^. Representative colonies of the transformants were used for 10-fold serial dilution, spotted on SDC (SDC-Leu-Trp), SDC-His (SDC-Leu-Trp-His), SDC-His+1 mM 3-AT (SDC-Leu-Trp-His+1 mM 3-AT) and SDC-Ade (SDC-Leu-Trp-Ade) plates. The plates were analyzed after 3 days growth at 30 °C. Successful transformation with both plasmids was confirmed by growth on SDC plates. The strength of interaction was usually assessed by growth on SDC-His (weak), SDC-His+1 mM 3-AT (medium) and SDC-Ade (strong). Controls for auto-activation were performed with empty pGADT7 plasmids. For the yeast three-hybrid experiments, a modified pRS426 plasmid (Ura3 selection) was generated, which contains the ADH1 promoter and an N-terminal NLS (nuclear localization sequence). This plasmid was used to express the bridging protein, or an empty version was used as a negative control. For the selection of triple co-transformants, SDC-Leu-Trp-Ura plates were used, and for binding assessment SDC-Leu-Trp-Ura-His plates were used. A full list of constructs generated and used in this study are listed in Supplementary Table 3.

### Multiple sequence alignment (MSA)

MSA was performed with Clustal Omega^50^, and ESPRIPT was used for visualization^51^. The ConSurf server^52^ was used to analyze conserved residues, and surface representation was generated with PyMOL (The PyMOL Molecular Graphics System, Version 1.8 Schrödinger, LLC).

### RNA extraction, reverse transcription, and qPCR

Total RNA from wt and mutant *S. pombe* strains was isolated using TriReagent (Sigma-Aldrich, T9424). Briefly, the cell pellet was resuspended in TriReagent and lysed at an OD_600_ of 8. Next the lysate was treated twice with 1-bromo-3-chloropropane (Sigma-Aldrich, B9673) followed by RNA precipitation using 2-propanol (Sigma-Aldrich, I9516). The RNA pellet was washed with ice-cold 75% ethanol and resuspended in nuclease-free water. The RNA concentration was measured using NanoDrop (Thermo Scientific) and 10 μg RNA was used for DNase treatment using DNaseI (NEB, M0303) following the manufacturer’s manual. The RNA samples were then reverse transcribed using Superscript III reverse transcriptase mix (Thermo Scientific,18080093). Random primers were used to obtain cDNA. Reverse transcription without reverse transcriptase (no RT control) was performed as the negative control. qPCR reactions were prepared using the Luna Universal qPCR master mix (NEB, M3003L) and run on QuantStudio 12k Flex System (Applied Biosciences). The qPCR data were analyzed with Microsoft Excel using the delta delta Ct method.

### Statistics and reproducibility

Figures 3b and 5a and Supplementary Figs. 1f, 2c, and 12b include representative SDS-PAGE gels from experiments which were performed at least two times.

### Reporting summary

Further information on research design is available in the Nature Research Reporting Summary linked to this article.

## Supplementary information

Supplementary Information

Reporting summary

## Data Availability

Accession codes: The coordinates and the structure factors have been deposited in the Protein Data Bank with the following accession code PDB ID: 6YGU (single-chain complex) and 6YFV (native complex). The PDB datasets 2XGJ, 4U4C, 6RO1, 5OOQ, and 6IEH have been used in this study. Protein sequences from Uniprot database with the following IDs were used in this study: G0RZ64, O13799, P42285, O14232, P47047, G0S1V1, Q9UTR8, V5IR63, B2RT41, O60293, G0SE05, O14253, G0S4F4, Q9P383, G0SBQ9, O94326, Q9BXP5-4, G0S5V0, Q9USP9, G0RZM1, O74823, G0S9J4, O14327, G0S6X0, and Q10295. Any other data supporting the findings of this study are available from the corresponding authors upon reasonable request. Source data are provided with this paper.

## Source data

Source Data
