# Supplementary material for: The zinc-finger protein Red1 orchestrates MTREC submodules and binds the Mtl1 helicase arch domain
Source: Nat Commun. 2021 Jun 8;12:3456. doi: 10.1038/s41467-021-23565-3 (PMC8187409; doi:10.1038/s41467-021-23565-3)
Supplement: Supplementary file 1 — Supplementary Information [file 41467_2021_23565_MOESM1_ESM.pdf]

SUPPLEMENTARY INFORMATION

The zinc-finger protein Red1 orchestrates MTREC submodules and binds the Mtl1 helicase arch domain

Nikolay Dobrev[1], Yasar Luqman Ahmed[1], Anusree Sivadas[2], Komal Soni[1], Tamás Fischer[1,2]*, Irmgard Sinning[1]*

[1]Heidelberg University Biochemistry Center (BZH), INF 328, D-69120 Heidelberg, Germany
[2]The John Curtin School of Medical Research, The Australian National University, Canberra, ACT 2601, Australia

* Correspondence should be addressed to
Irmgard Sinning (irmi.sinning@bzh.uni-heidelberg.de) or
Tamás Fischer (tamas.fischer@anu.edu.au)

Content:

Supplementary figures 1 to 15
Supplementary tables 1 to 3
Supplementary references 1 to 13

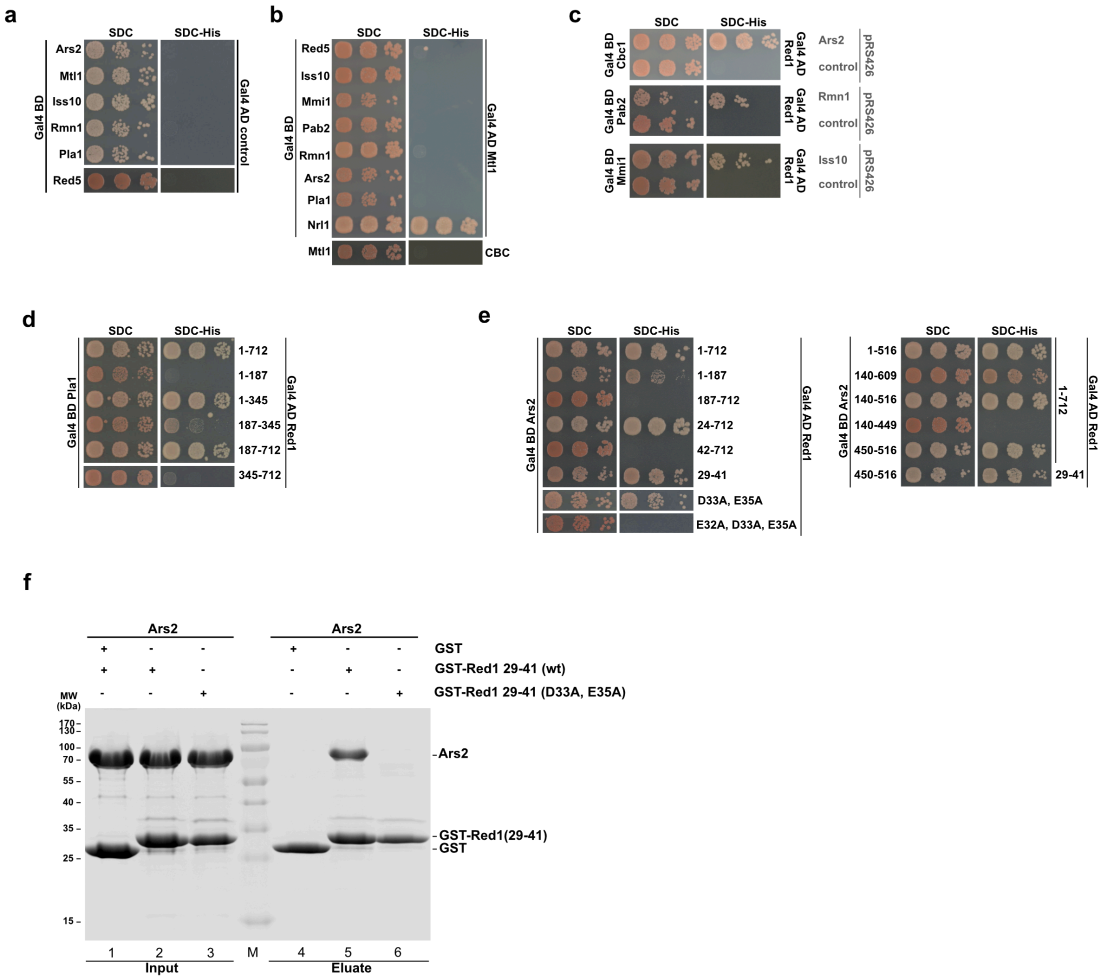

Supplementary Figure 1 Analysis of interactions between MTREC components

a Auto-activation controls for the interactions presented in Fig. 1b. **b** Y2H experiments showing no interaction between Mtl1 and the other MTREC subunits. Nrl1 is used as positive control. Nrl1 interacts with Mtl1 and Ctr1 to form the CNM complex[1,2]. **c** Ars2 bridges Cbc1 and Red1 in Y3H analysis. Similarly, Pab2 and Mmi1 interact with Red1 in presence of Rmn1 and Iss10, respectively. **d** Pla1 interacts with various Red1 truncation variants**.** **e** Ars2 interacts with various Red1 truncation variants (left panel), and Ars2 truncation variants with Red1 (right panel)**.** **f** Coomassie stained SDS-PAGE of an *in vitro* GST pull-down assay with the Red1 peptide (residues 29 to 41) and Ars2. The Red1 double mutant (D33A, E35A) does not interact with Ars2.

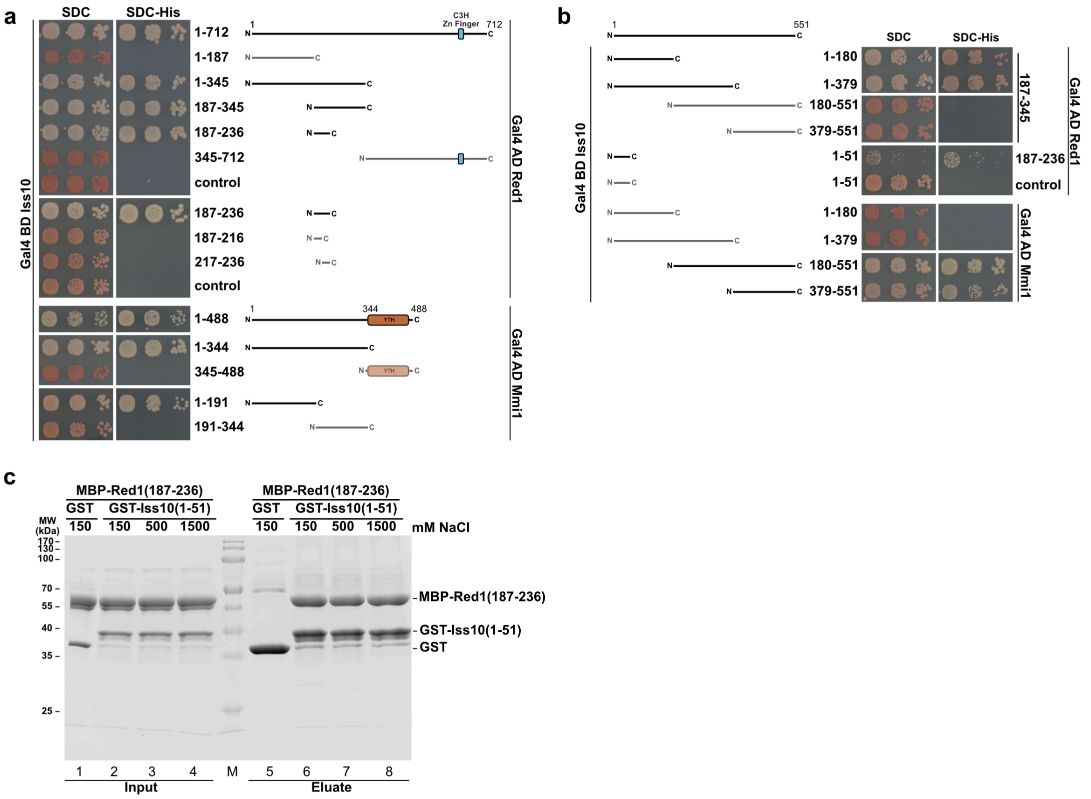

Supplementary Figure 2 Analysis of interactions between the Mmi1-Iss10 submodule and Red1

a, b Y2H analysis of the Mmi1-Iss10 and Red1 interaction. Using Red1, Iss10 and Mmi1 truncation variants, the interacting regions were narrowed down to Iss10 (residues 379 to 551) and Mmi1 (residues 1 to 191) in the Mmi1-Iss10 submodule, and to Red1 (residues 187 to 236) and Iss10 (residues 1 to 51). (c) Coomassie stained SDS-PAGE of an *in vitro* GST pull-down assay with MBP-Red1 (residues 187 to 236) and GST-Iss10 (residues 1 to 51). The Iss10 N-terminal region interacts with the Red1 truncation variant, and this interaction is stable under high salt conditions (1.5 M NaCl).

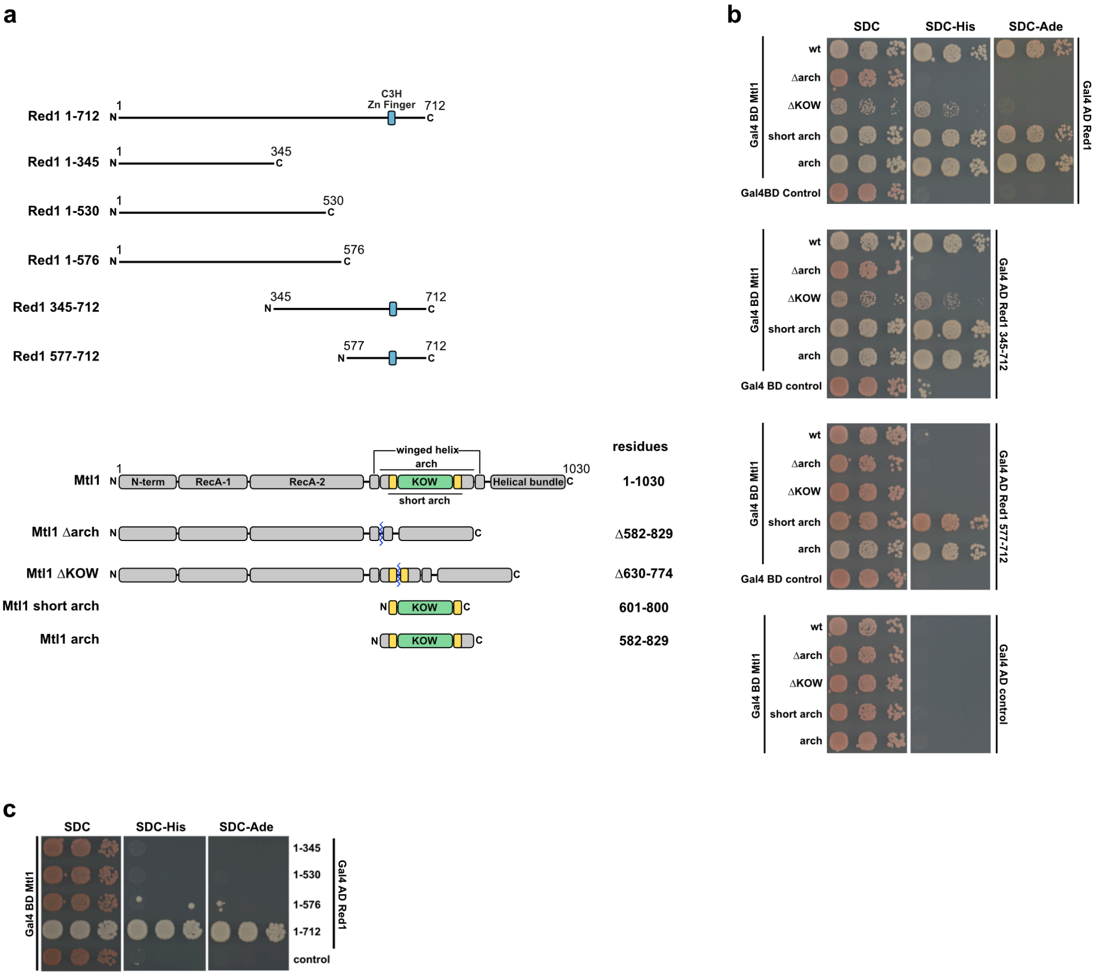

Supplementary Figure 3 Analysis of interactions between Red1 and Mtl1

a Scheme of Red1 and Mtl1 variants used in Y2H analyses. **b** Y2H analysis of Mtl1 and Red1 truncation variants. The Mtl1 ΔKOW domain shows much weaker interaction with Red1 compared to the arch domain indicated by slow growth on SDC-His and no growth on SDC-Ade. **c** Y2H analysis of Mtl1 and Red1 N-terminal truncation variants. Gal4 BD, DNA binding domain; Gal4 AD, activation domain; SDC-Leu-Trp (SDC), SDC-Leu-Trp-His (SDC-His) and SDC-Leu-Trp-Ade(SDC-Ade).

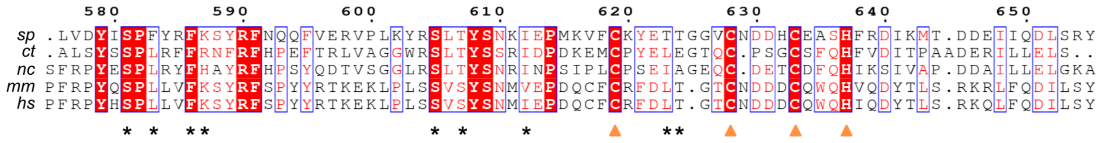

Supplementary Figure 4 Multiple sequence alignment of the conserved Red1 C-terminal region

Sequences from *Chaetomium thermophilum* (*ct)* – *ct*Red1 (Uniprot ID: G0S1V1, see Supplementary Figure 12), *Schizosaccharomyces pombe* (*sp*) – *sp*Red1 (Uniprot ID: Q9UTR8), *Neurospora crassa* (*nc*) – *nc*Red1 (Uniprot ID: V5IR63), *Mus musculus* (*mm*) – *mmZfc3h1* (Uniprot ID: B2RT41) and *Homo sapiens* (*hs*) - *hsZFC3H1 (Uniprot ID: O60293)*. Fully conserved residues are highlighted in red. Residues selected for mutational analysis are marked with an asterisk. Orange arrowheads indicate residues involved in Zinc-finger formation.

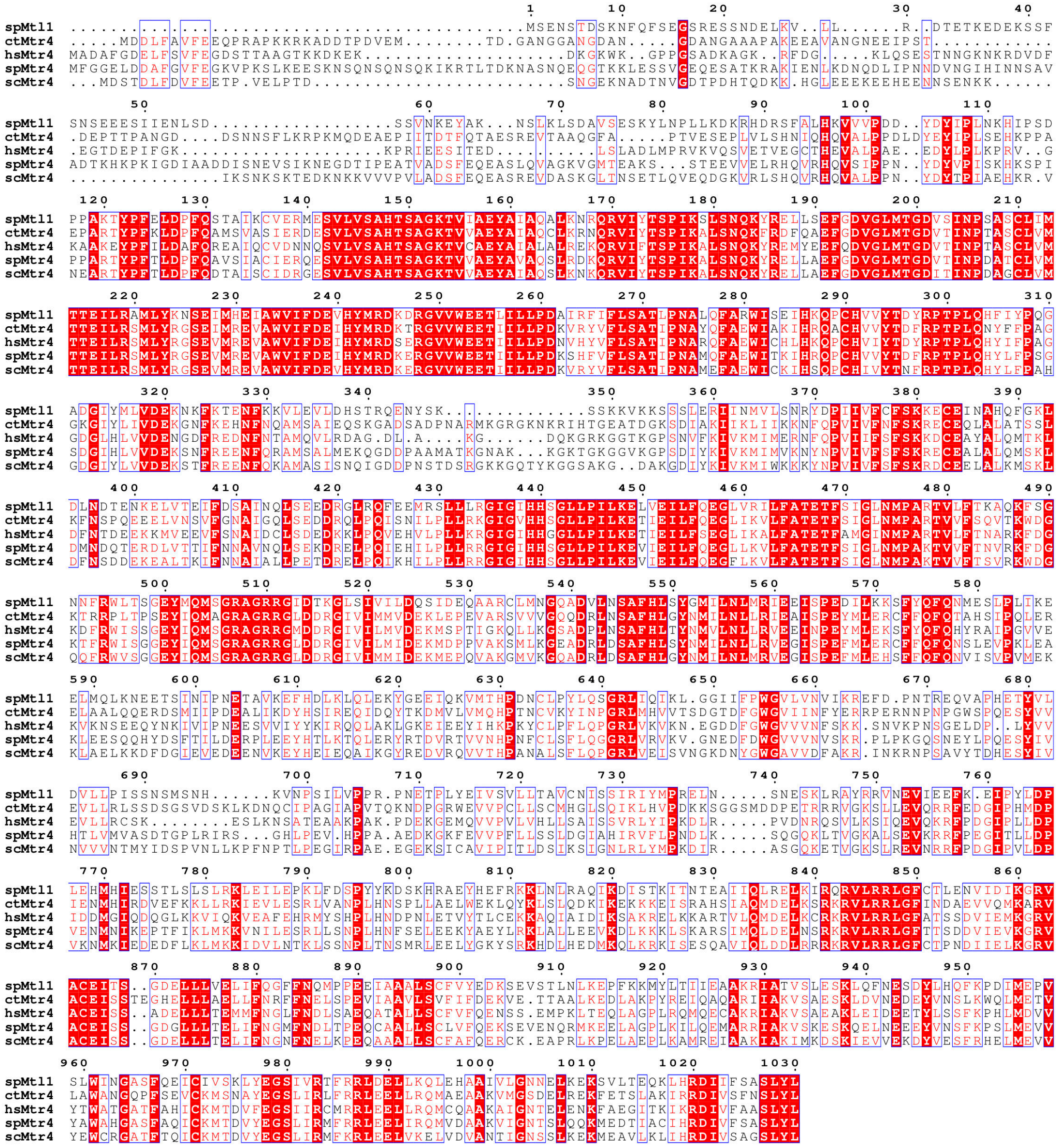

Supplementary Figure 5 Sequence alignment of Mtr4/Mtl1

Multiple sequence alignment of *Chaetomium thermophilum* Mtr4 (*ct*Mtr4, Uniprot ID: G0RZ64) with corresponding region of *Schizosaccharomyces pombe* Mtl1 (*sp*Mtl1*, Uniprot ID:* O13799), *Homo sapiens* Mtr4 (*hs*Mtr4*, Uniprot ID:* P42285), *Schizosaccharomyces pombe* Mtr4 (*sp*Mtr4*, Uniprot ID:* O14232) and *Saccharomyces cerevisiae* Mtr4 (*sc*Mtr4, Uniprot ID: P47047). Fully conserved residues are highlighted in red.

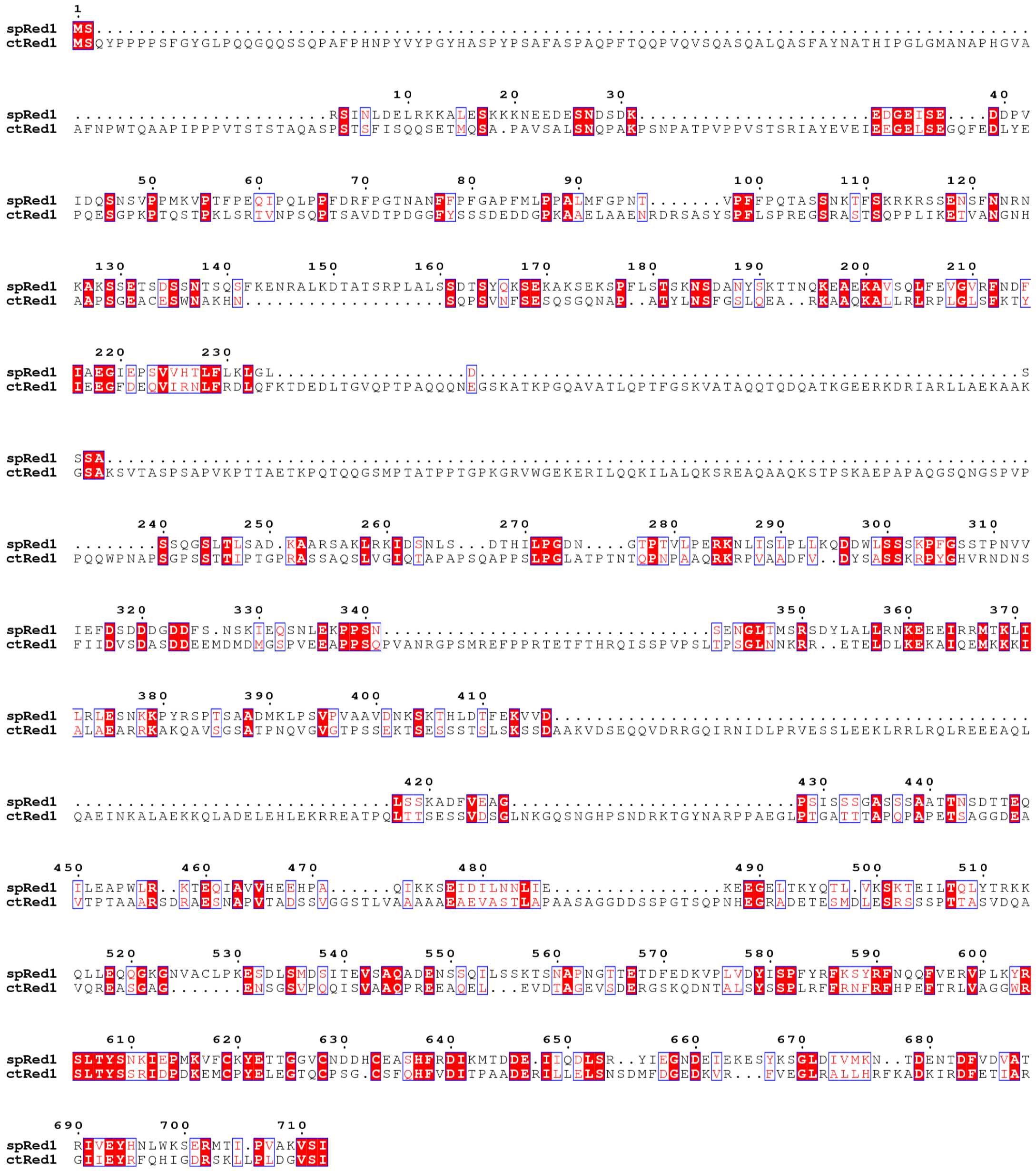

Supplementary Figure 6 Sequence alignment of Red1 from *Chaetomium thermophilum* and *Schizosaccharomyces pombe*

Sequence alignment of *ct*Red1(Uniprot ID: G0S1V1, see Supplement figure 12) and *sp*Red1 (Uniprot ID: Q9UTR8).

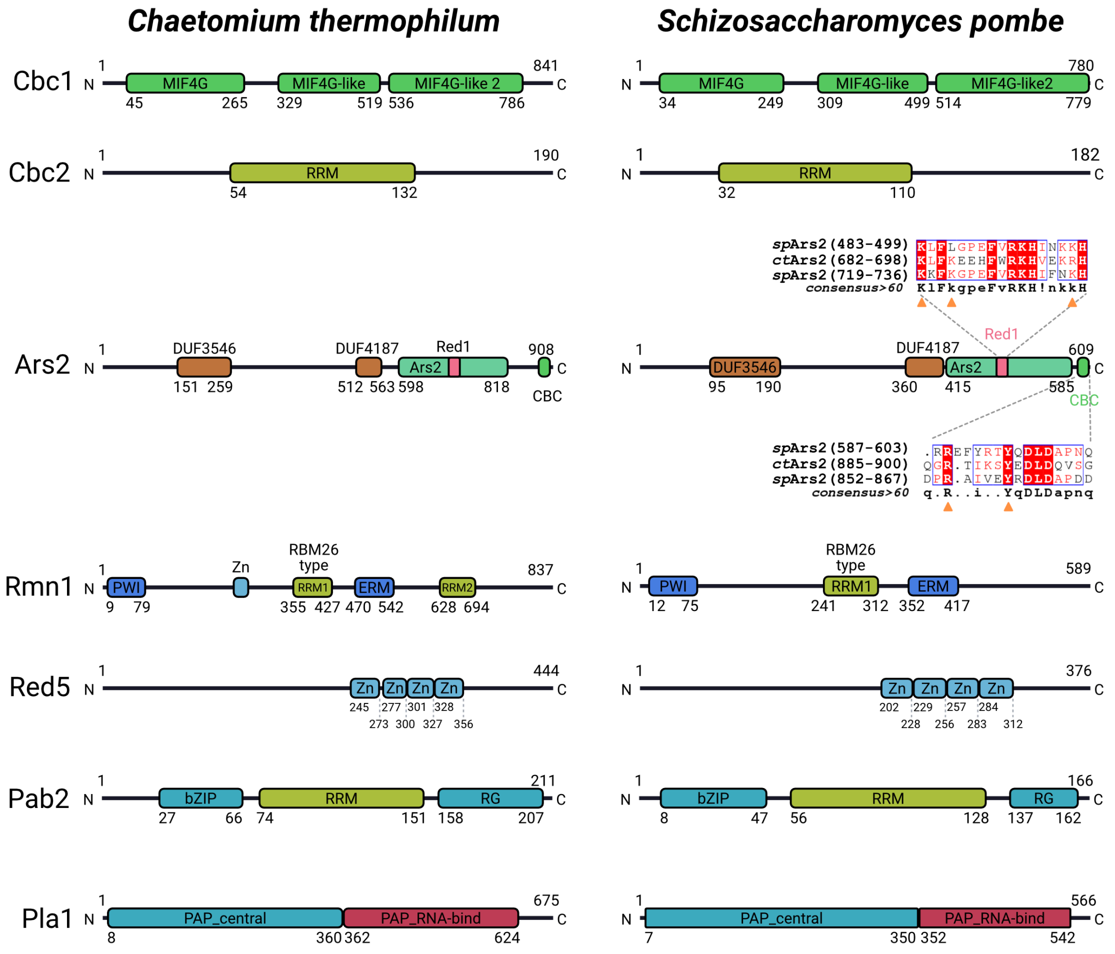

Supplementary Figure 7 Domain organization of MTREC components in of *Chaetomium thermophilum* and *Schizosaccharomyces pombe*

The domain organization of MTREC proteins of *Chaetomium thermophilum* and *Schizosaccharomyces pombe* is shown. *ct*Cbc1 (Uniprot ID: G0SE05) and *sp*Cbc1 (Uniprot ID: O14253); *ct*Cbc2 (Uniprot ID: G0S4F4) and *sp*Cbc2 (Uniprot ID: Q9P383); *ct*Ars2 (Uniprot ID: G0SBQ9) and *sp*Ars2 (Uniprot ID: O94326) – partial alignments of the Red1 and CBC interaction regions are shown. Orange arrowheads indicate residues previously shown to be critical for binding FLASH (PMID: 29703953) and CBC (PMID: 29101316), respectively. *Hs*Ars2 (Uniprot ID: Q9BXP5-4) was used for the alignment; *ct*Rmn1 (Uniprot ID: G0S5V0) and *sp*Rmn1 (Uniprot ID: Q9USP9); *ct*Red5 (Uniprot ID: G0RZM1) and *sp*Red5 (Uniprot ID: O74823); *ct*Pab2 (Uniprot ID: G0S9J4) and *sp*Pab2 (Uniprot ID: O14327); *ct*Pla1 (Uniprot ID: G0S6X0) and *sp*Pla1 (Uniprot ID: Q10295).

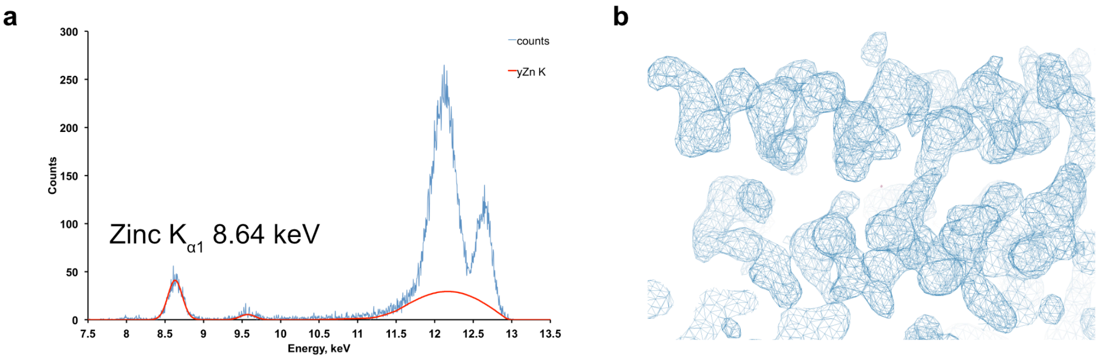

Supplementary Figure 8 X-ray structure determination using Zn-SAD

a X-ray fluorescence (XRF) spectrum revealing the presence of Zn^{2+} in the *ct*Mtr4-*ct*Red1 crystal. **b** Initial composite (2Fo - Fc, contoured at 1.5 σ) electron density map. The figure was made in *Coot*[3].

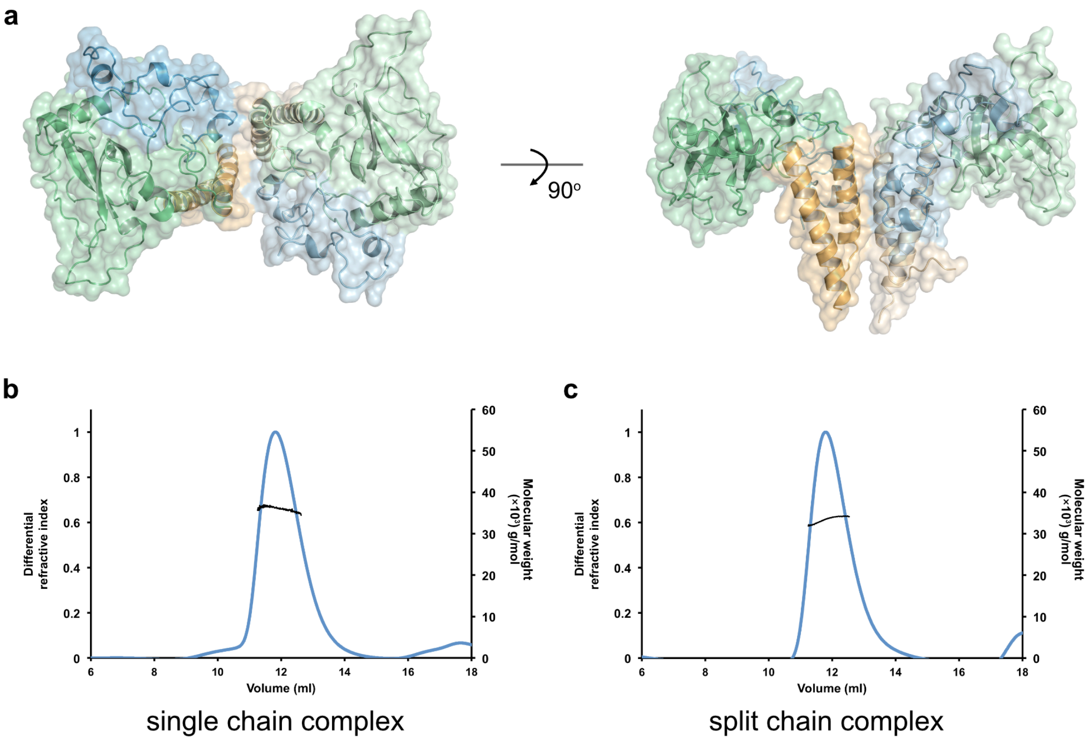

Supplementary Figure 9 Analysis of the *ct*Mtr4$_{SA}$-*ct*Red1$_{pep}$ complex

a The single-chain *ct*Mtr4$_{SA}$-*ct*Red1$_{pep}$ complex crystallizes with a crystallographic dimer in the ASU. The dimer is shown in cartoon and surface representation. The color scheme is as follows: *ct*Red1$_{pep}$ (blue), *ct*Mtr4 KOW domain (green) and stalk helices (orange). **b, c** SEC-MALS analysis of the oligomerization state for the single-chain complex (in b) and the split-chain complex (in c). Both complexes show a molecular weight of ~34 kDa indicating a monomer in solution.

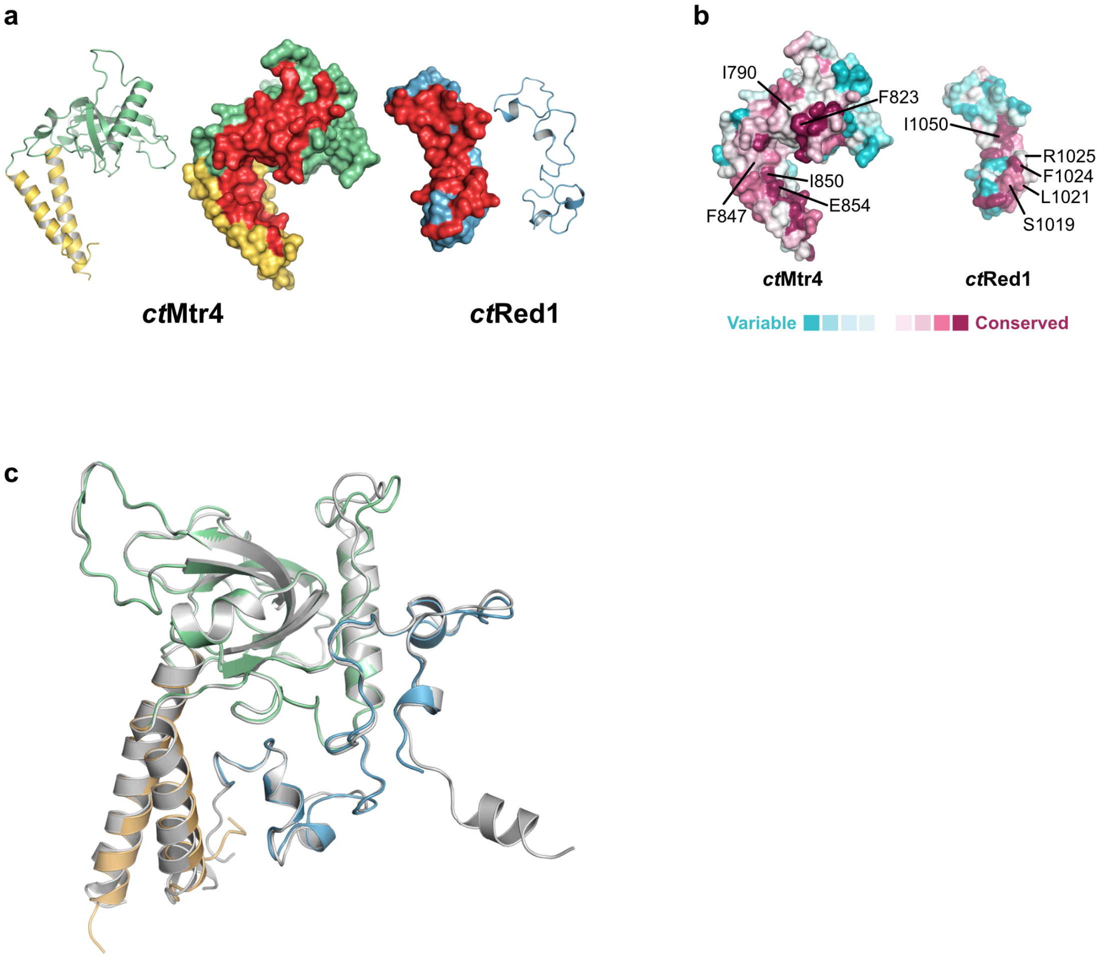

Supplementary Figure 10 Conservation analysis of the *ct*Mtr4$_{SA}$-*ct*Red1$_{pep}$ interface

a Interaction between *ct*Mtr4$_{SA}$ and *ct*Red1$_{pep}$ involves a large surface area of 1319.8 Å^2 (*ct*Mtr4$_{SA}$) and 1403.2 Å^2 (*ct*Red1$_{pep}$). Both proteins are shown side by side in cartoon and surface representation. Residues involved in the interaction are highlighted (red) in the surface representation. **b** The interacting surfaces of both proteins are shown colored by conservation (analyzed with ConSurf webserver[4]). The majority of residues in the interface are conserved. Variable amino acids are shown in mint-green and conserved amino acids in red. **c** Superposition of single chain (in color) and split chain complex (gray) shows only small differences.

<table>
<tr><th>ctMtr4</th><th>HSDC</th><th>ASA</th><th>BSA</th><th>ΔiG</th></tr>
<tr><td>A:TYR 661</td><td></td><td>35.95</td><td>1.60 |</td><td>-0.02</td></tr>
<tr><td>A:ARG 665</td><td></td><td>76.40</td><td>20.18 |||</td><td>-0.09</td></tr>
<tr><td>A:ILE 668</td><td></td><td>10.97</td><td>8.46 ||||||||</td><td>0.14</td></tr>
<tr><td>A:THR 672</td><td></td><td>50.50</td><td>19.93 ||||</td><td>0.12</td></tr>
<tr><td>A:MET 675</td><td></td><td>36.23</td><td>31.55 |||||||||</td><td>0.76</td></tr>
<tr><td>A:MET 679</td><td></td><td>37.15</td><td>11.25 ||||</td><td>0.28</td></tr>
<tr><td>A:TYR 688</td><td></td><td>26.61</td><td>0.31 |</td><td>0.01</td></tr>
<tr><td>A:ASN 690</td><td></td><td>38.97</td><td>1.85 |</td><td>-0.01</td></tr>
<tr><td>A:PRO 691</td><td></td><td>77.01</td><td>2.83 |</td><td>-0.03</td></tr>
<tr><td>A:ARG 693</td><td>HS</td><td>26.79</td><td>17.59 |||||||</td><td>-0.59</td></tr>
<tr><td>A:GLN 789</td><td></td><td>87.83</td><td>77.05 |||||||||</td><td>-0.48</td></tr>
<tr><td>A:ILE 790</td><td></td><td>42.51</td><td>42.34 ||||||||||</td><td>0.68</td></tr>
<tr><td>A:LYS 791</td><td>H</td><td>110.36</td><td>85.46 ||||||||</td><td>-0.00</td></tr>
<tr><td>A:LEU 792</td><td></td><td>31.54</td><td>27.68 |||||||||</td><td>0.34</td></tr>
<tr><td>A:HIS 793</td><td>HS</td><td>138.42</td><td>81.65 ||||||</td><td>-0.43</td></tr>
<tr><td>A:VAL 794</td><td></td><td>18.08</td><td>2.15 ||</td><td>-0.00</td></tr>
<tr><td>A:ASP 796</td><td></td><td>62.42</td><td>3.31 |</td><td>-0.06</td></tr>
<tr><td>A:ARG 811</td><td></td><td>147.59</td><td>30.09 |||</td><td>0.16</td></tr>
<tr><td>A:SER 815</td><td></td><td>47.05</td><td>29.58 |||||||</td><td>0.43</td></tr>
<tr><td>A:GLU 818</td><td></td><td>72.70</td><td>31.85 |||||</td><td>0.44</td></tr>
<tr><td>A:VAL 819</td><td></td><td>25.99</td><td>25.99 |||||||||||</td><td>0.40</td></tr>
<tr><td>A:ARG 822</td><td></td><td>178.68</td><td>90.52 ||||||</td><td>-0.92</td></tr>
<tr><td>A:PHE 823</td><td></td><td>69.93</td><td>64.84 ||||||||||</td><td>1.04</td></tr>
<tr><td>A:GLU 824</td><td></td><td>162.09</td><td>24.83 ||</td><td>-0.23</td></tr>
<tr><td>A:ASP 825</td><td>H</td><td>138.18</td><td>106.54||||||||</td><td>-0.38</td></tr>
<tr><td>A:GLY 826</td><td>H</td><td>40.60</td><td>19.03 |||||</td><td>0.08</td></tr>
<tr><td>A:ILE 827</td><td></td><td>13.29</td><td>5.39 |||||</td><td>-0.06</td></tr>
<tr><td>A:PRO 828</td><td></td><td>34.31</td><td>33.97 ||||||||||</td><td>0.54</td></tr>
<tr><td>A:HIS 829</td><td></td><td>95.46</td><td>72.06 ||||||||</td><td>0.71</td></tr>
<tr><td>A:MET 830</td><td></td><td>15.31</td><td>11.82 ||||||||</td><td>0.24</td></tr>
<tr><td>A:ASP 831</td><td>H</td><td>57.75</td><td>49.58 |||||||||</td><td>-0.27</td></tr>
<tr><td>A:PRO 832</td><td></td><td>19.27</td><td>18.60 ||||||||||</td><td>0.30</td></tr>
<tr><td>A:ILE 833</td><td></td><td>49.56</td><td>4.69 |</td><td>0.08</td></tr>
<tr><td>A:GLU 834</td><td>HS</td><td>134.89</td><td>53.46 ||||</td><td>-0.56</td></tr>
<tr><td>A:ASN 835</td><td></td><td>48.37</td><td>12.50 |||</td><td>-0.13</td></tr>
<tr><td>A:LEU 847</td><td></td><td>45.06</td><td>25.50 ||||||</td><td>0.38</td></tr>
<tr><td>A:ILE 850</td><td></td><td>20.04</td><td>20.04 |||||||||||</td><td>0.32</td></tr>
<tr><td>A:GLU 851</td><td></td><td>92.65</td><td>39.18 |||||</td><td>-0.29</td></tr>
<tr><td>A:GLU 854</td><td>H</td><td>81.65</td><td>59.42 ||||||||</td><td>-0.51</td></tr>
<tr><td>A:LEU 857</td><td></td><td>14.39</td><td>14.39 |||||||||||</td><td>0.23</td></tr>
<tr><td>A:VAL 858</td><td></td><td>90.36</td><td>5.52 |</td><td>0.09</td></tr>
<tr><td>A:HIS 863</td><td></td><td>73.35</td><td>26.48 ||||</td><td>0.17</td></tr>
<tr><td>A:ASN 864</td><td></td><td>172.25</td><td>2.34 |</td><td>0.04</td></tr>
</table>

<table>
<tr><th>ctRed1</th><th>HSDC</th><th>ASA</th><th>BSA</th><th>ΔiG</th></tr>
<tr><td>B:TYR1017</td><td>H</td><td>243.57</td><td>128.06 ||||||</td><td>1.23</td></tr>
<tr><td>B:SER1019</td><td>H</td><td>34.19</td><td>16.56 |||||</td><td>0.06</td></tr>
<tr><td>B:PRO1020</td><td></td><td>98.90</td><td>64.14 |||||||</td><td>0.96</td></tr>
<tr><td>B:LEU1021</td><td></td><td>107.77</td><td>93.35 |||||||||</td><td>1.49</td></tr>
<tr><td>B:PHE1023</td><td></td><td>124.21</td><td>77.76 |||||||</td><td>0.88</td></tr>
<tr><td>B:PHE1024</td><td></td><td>127.67</td><td>109.62 |||||||||</td><td>1.75</td></tr>
<tr><td>B:ARG1025</td><td>HS</td><td>111.45</td><td>71.49 |||||||</td><td>0.41</td></tr>
<tr><td>B:ASN1026</td><td></td><td>119.99</td><td>36.19 ||||</td><td>-0.04</td></tr>
<tr><td>B:ARG1028</td><td></td><td>37.69</td><td>4.45 ||</td><td>-0.05</td></tr>
<tr><td>B:VAL1037</td><td></td><td>21.87</td><td>10.54 |||||</td><td>0.17</td></tr>
<tr><td>B:ALA1038</td><td>H</td><td>110.60</td><td>30.18 |||</td><td>0.40</td></tr>
<tr><td>B:GLY1039</td><td>H</td><td>26.74</td><td>12.62 |||||</td><td>0.06</td></tr>
<tr><td>B:SER1043</td><td>H</td><td>13.72</td><td>9.53 |||||||</td><td>-0.09</td></tr>
<tr><td>B:LEU1044</td><td></td><td>69.57</td><td>31.49 |||||</td><td>0.45</td></tr>
<tr><td>B:THR1045</td><td>H</td><td>82.45</td><td>78.34 ||||||||||</td><td>-0.06</td></tr>
<tr><td>B:TYR1046</td><td></td><td>17.97</td><td>1.38 |</td><td>0.02</td></tr>
<tr><td>B:SER1047</td><td></td><td>13.46</td><td>10.42 ||||||||</td><td>-0.12</td></tr>
<tr><td>B:SER1048</td><td></td><td>79.03</td><td>0.49 |</td><td>0.01</td></tr>
<tr><td>B:ILE1050</td><td></td><td>80.74</td><td>69.28 |||||||||</td><td>0.97</td></tr>
<tr><td>B:ASP1051</td><td></td><td>55.61</td><td>0.98 |</td><td>-0.01</td></tr>
<tr><td>B:PRO1052</td><td>H</td><td>124.34</td><td>80.41 |||||||</td><td>0.53</td></tr>
<tr><td>B:ASP1053</td><td>HS</td><td>130.89</td><td>29.22 |||</td><td>-0.09</td></tr>
<tr><td>B:LYS1054</td><td></td><td>85.44</td><td>7.85 |</td><td>-0.04</td></tr>
<tr><td>B:GLU1055</td><td>HS</td><td>122.99</td><td>76.89 |||||||</td><td>0.35</td></tr>
<tr><td>B:MET1056</td><td>H</td><td>52.72</td><td>52.07 ||||||||||</td><td>0.65</td></tr>
<tr><td>B:PRO1058</td><td></td><td>58.79</td><td>26.89 |||||</td><td>0.42</td></tr>
<tr><td>B:GLU1060</td><td></td><td>71.90</td><td>19.67 |||</td><td>-0.03</td></tr>
<tr><td>B:LEU1061</td><td></td><td>119.07</td><td>107.68 ||||||||||</td><td>1.15</td></tr>
<tr><td>B:GLU1062</td><td></td><td>122.71</td><td>38.38 ||||</td><td>-0.25</td></tr>
<tr><td>B:PHE1072</td><td></td><td>64.69</td><td>14.84 |||</td><td>0.24</td></tr>
<tr><td>B:GLN1073</td><td></td><td>6.06</td><td>1.02 ||</td><td>-0.01</td></tr>
<tr><td>B:PHE1075</td><td></td><td>81.25</td><td>72.99 |||||||||</td><td>1.17</td></tr>
<tr><td>B:ILE1078</td><td></td><td>8.87</td><td>8.87 ||||||||||</td><td>0.14</td></tr>
</table>

Supplementary Figure 11 PISA analysis of *ct*Mtr4$_{SA}$-*ct*Red1$_{pep}$ interface Analysis of the *ct*Mtr4-*ct*Red1 interaction observed in the crystal structure with the PISA webserver[5] (**HSDC** (Residues making **H**ydrogen/**D**isulphide bond, **S**alt bridge or **C**ovalent); **ASA** Accessible Surface Area, Å²; **BSA** Buried Surface Area, Å². The bars represented the buried area percentage, one bar per 10% ; **ΔiG** Solvation energy effect, kcal/mol.

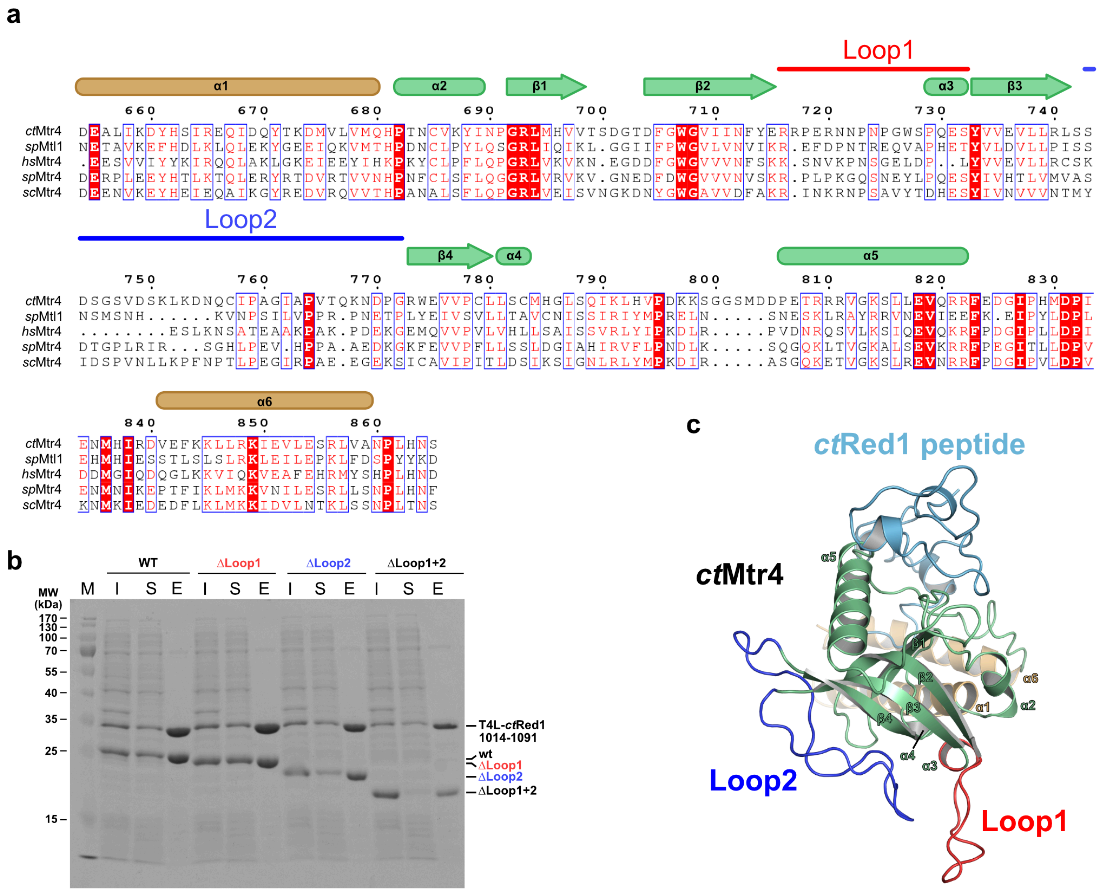

Supplementary Figure 12 Deletion of *ct*Mtr4 KOW domain loops 1 and 2 does not perturb *ct*Red1 binding

a Multiple sequence alignment of *Chaetomium thermophilum* Mtr4$_{SA}$ (*ct*Mtr4, Uniprot ID: G0RZ64) with corresponding region of *Schizosaccharomyces pombe* Mtl1 (*sp*Mtl1*, Uniprot ID:* O13799), *Homo sapiens* Mtr4 (*hs*Mtr4*, Uniprot ID:* P42285), *Schizosaccharomyces pombe* Mtr4 (*sp*Mtr4*, Uniprot ID:* O14232) and *Saccharomyces cerevisiae* Mtr4 (*sc*Mtr4, Uniprot ID: P47047). Fully conserved residues are highlighted in red. The secondary structure is given above the alignment, with stalk helices shown in orange and the KOW domain in green. The KOW domain loops 1 and 2 are highlighted in red and blue. **b** Coomassie stained SDS-PAGE of His$_6$-tagged T4L-*ct*Red1$_{pep}$ co-expressed and purified with untagged *ct*Mtr4$_{SA\Delta loop1}$, *ct*Mtr4$_{SA\Delta loop2}$ and *ct*Mtr4$_{SA\Delta loop1+2}$. Removal of loop1, loop2 or both does not abolish binding to *ct*Red1$_{pep}$. **c** Ribbon representation of the *ct*Mtr4$_{SA}$-*ct*Red1$_{pep}$ structure with loop1 (red) and loop2 (blue) highlighted.

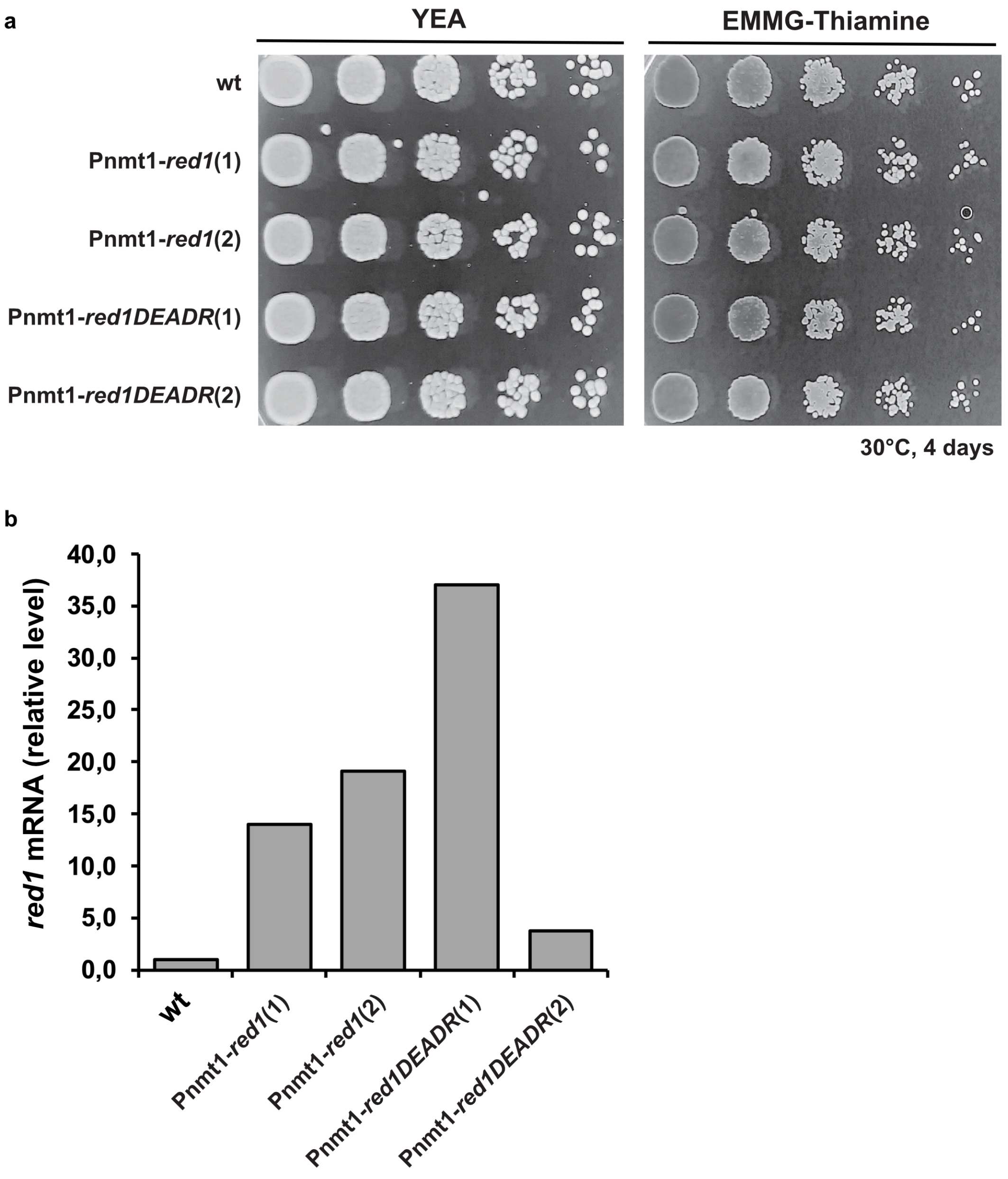

Supplementary Figure 13 *red1 DEADR* mutation is not a dominant negative allele

Pnmt1-*red1* and Pnmt1-*red1 DEADR* alleles were inserted in the Leu1 locus of wt *S. pombe* genome and (**a**) 5-fold serial dilution of 2 independent strains (labeled with 1 and 2) of each were spotted on YEA media (Pnmt1 promoter is repressed, non-overexpressing condition) and on EMMG-Thiamine media (Pnmt1 promoter is strongly induced, overexpressing condition). (**b**) qPCR analysis of the *red1* mRNA expression level for these strains in EMMG-Thiamine media, compared to *red1* expression in wt *S. pombe* cells. Two biological replicates were performed (labeled with 1 and 2), n = 2. Source data are provided as a Source Data file.

a

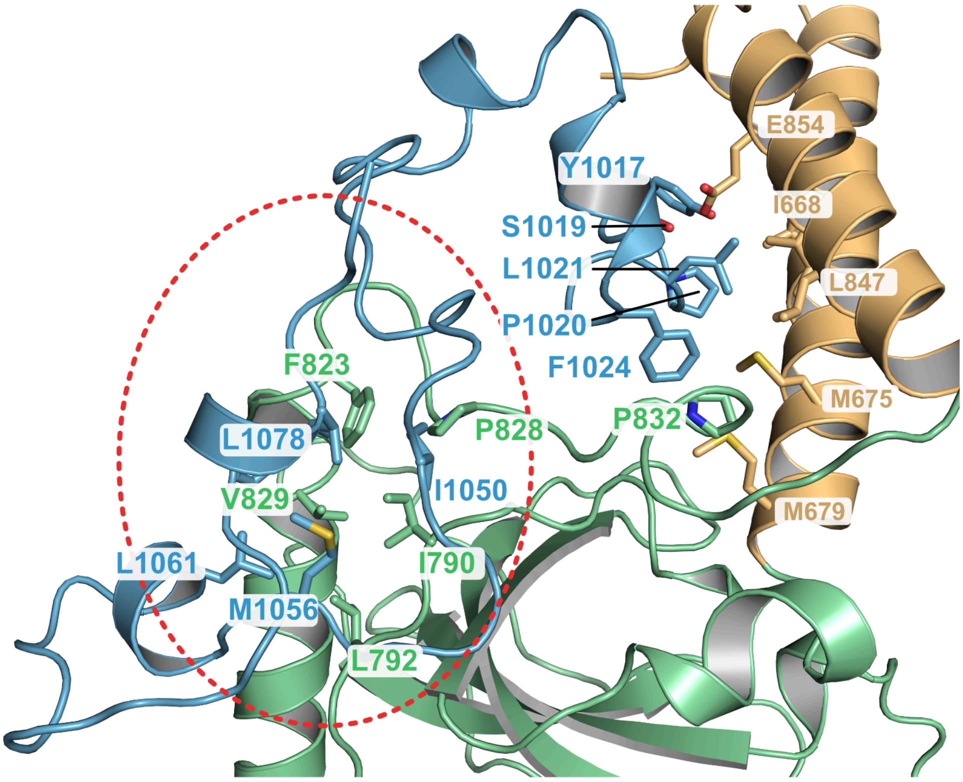

b

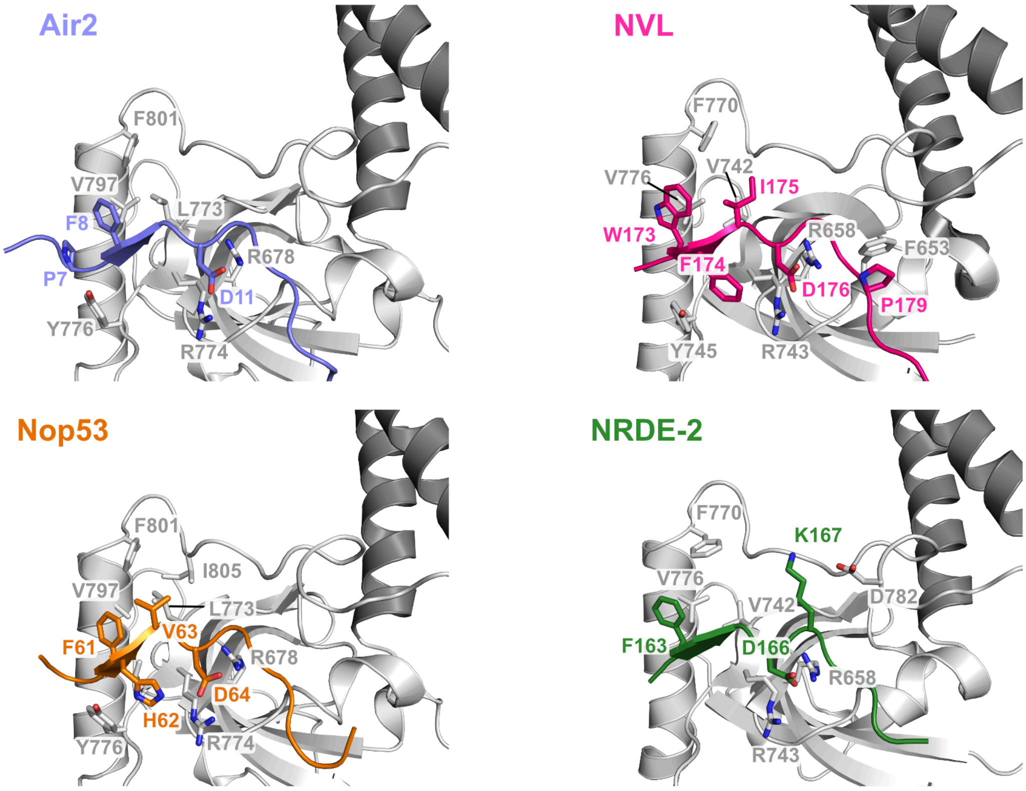

Supplementary Figure 14 Detailed comparison of the *ct*Mtr4-*ct*Red1 crystal structure with known Mtr4 complexes

a Crystal structure of the *ct*Mtr4$_{SA}$-*ct*Red1$_{pep}$ complex (colors are as in Fig. 4). Residues involved in the interaction between *ct*Mtr4-*ct*Red1 are shown in sticks

and the binding interface of the AIM or AIM-like adaptor proteins is highlighted by a red dotted line. **b** The structures of Mtr4-Air2 (PDB: 4U4C[6] top left), Mtr4-NVL (PDB: 6RO1[7] top right), Mtr4-Nop53 (PDB: 5OOQ[8] bottom left) and hMTR4-NRDE-2 (PDB: 6IEH[9] bottom right), are shown (same view as in **a**). The stalk helices are shown in dark grey, the KOW domain is in light grey and the interacting peptides are shown in purple (Air2), magenta (NVL), orange (Nop53) and green (NRDE-2). The residues involved in the interaction are shown as sticks.

```
           980       990       1000      1010      1020      1030
G0S1V1  QISVAAQPREEAQELEVDTAGEVKVVSRGGVDTDQLTDHQVSDERGSKQDNTALSYSSPL
cDNA    QISVAAQPREEAQELEVDTAGE..................VSDERGSKQDNTALSYSSPL

          1040      1050      1060      1070      1080      1090
G0S1V1  RFFRNFRFHPEFTRLVAGGWRSLTYSSRIDPDKEMCPYELEGTQCPSGCSFQHFVDITPA
cDNA    RFFRNFRFHPEFTRLVAGGWRSLTYSSRIDPDKEMCPYELEGTQCPSGCSFQHFVDITPA

           1100      1110      1120      1130      1140      1150
G0S1V1  .DERILLELSNSDMFDGEDKVRFVEGLRALLHRFKADKIRDFETIARGIIEYRFQHIGDR
cDNA    ADERILLELSNSDMFDGEDKVRFVEGLRALLHRFKADKIRDFETIARGIIEYRFQHIGDR

           1160
G0S1V1  SKLLPLDGVSI
cDNA    SKLLPLDGVSI
```

Supplementary Figure 15 Sequence alignment of the conserved Red1 C-terminal region

The *ct*Red1 C-terminal region from the Uniprot ID G0S1V1 sequence is compared with the sequence amplified from cDNA. Differences are highlighted by red boxes, which leads to differences in residue numbers. The sequence of our crystallization construct is indicated in blue. Differences between "observed" protein sequences and those annotated in the *Chaetomium thermophilum* genome database have been noticed in the past years and await correction. The *ct*Red1 sequence adds to this list.

Supplementary Table 1. Yeast strain used in this study

Nr.	Name	Relevant Genotype	Reference
S. pombe			
P344	WT	*h+, leu1-32, ura4D18, ade6-M210*	V2-33-H12, Bioneer Inc
P918	WT	*h90, leu1-32, ura4D18, ade6-M210*	Sugiyama et al (2011)[10]
P934	red1Δ	*h90, leu1-32, ura4D18, ade6-M210, red1Δ::kanMX6*	Sugiyama et al (2011)[10]
F3091	Mtl1(L784R/E788R) (E1)	*h90, leu1-32, ura4D18, ade6-M210, mtl1-L784R/E788R::natNT2*	this study
F3092	Mtl1(WT) (E2)	*h90, leu1-32, ura4D18, ade6-M210*	this study
F3093	Mtl1(L784R/E788R) (E3)	*h90, leu1-32, ura4D18, ade6-M210, mtl1-L784R/E788R::natNT2*	this study
F3094	Mtl1(L784R/E788R) (B1)	*h90, leu1-32, ura4D18, ade6-M210, mtl1-L784R/E788R::natNT2*	this study
F3095	Mtl1(WT) (B2)	*h90, leu1-32, ura4D18, ade6-M210*	this study
F3096	Mtl1(L784R/E788R) (B3)	*h90, leu1-32, ura4D18, ade6-M210, mtl1-L784R/E788R::natNT2*	this study
NP70	Red1(DEADR) (A1)	*h90, leu1-32, ura4D18, ade6-M210, red1-DEADR::hphNT1*	this study
NP71	Red1(DEADR) (A4)	*h90, leu1-32, ura4D18, ade6-M210, red1-DEADR::hphNT1*	this study
NP72	Red1(DEADR) (B2)	*h90, leu1-32, ura4D18, ade6-M210, red1-DEADR::hphNT1*	this study
NP73	Red1(WT) (B3)	*h90, leu1-32, ura4D18, ade6-M210*	this study
NP74	Red1(DEADR) (B4)	*h90, leu1-32, ura4D18, ade6-M210, red1-DEADR::hphNT1*	this study

NP75	Red1(DEADR) (D1)	*h90, leu1-32, ura4D18, ade6-M210, red1-DEADR::hphNT1*	this study
NP76	Red1(DEADR) (D2)	*h90, leu1-32, ura4D18, ade6-M210, red1-DEADR::hphNT1*	this study
NP77	Red1(WT) (B3)	*h90, leu1-32, ura4D18, ade6-M210*	this study
NP78	red1∆ (B4)	*h90, leu1-32, ura4D18, ade6-M210, red1∆::kanMX6*	this study
NP79	red1∆ (E4)	*h90, leu1-32, ura4D18, ade6-M210, red1∆::kanMX6*	this study
F3321	Pnmt1-Red1 (1)	*h+, HIS, leu1-32, ade6-210, ura4-D18, 5'Leu1-pnmt1-red1-3'Leu1::kanMX6*	this study
F3320	Pnmt1-Red1 (2)	*h+, HIS, leu1-32, ade6-210, ura4-D18, 5'Leu1-pnmt1-red1-3'Leu1::kanMX6*	this study
F3322	Pnmt1-Red1DEADR (1)	*h+, HIS, leu1-32, ade6-210, ura4-D18, 5'Leu1-pnmt1-red1-DEADR-3'Leu1::kanMX6*	this study
F3323	Pnmt1-Red1DEADR (2)	*h+, HIS, leu1-32, ade6-210, ura4-D18, 5'Leu1-pnmt1-red1-DEADR-3'Leu1::kanMX6*	this study
S. cerevisiae			
S145	PJ69-4A	MATα, trp1-901, leu2-3,112, ura3-52, his3-200, gal4Δ, gal80Δ, LYS2::GAL1-HIS3, GAL2-ADE2, met2::GAL7-lacZ	James et al., 1996[11]

Supplementary Table 2. Primers used in this study

Nr.	Name	Sequence (5'->3')
**N1**	*sp*Cbc1_FW_NcoI	CACCATCACCATCACCCCATGGCTTCTTATCGGGGATCAA
**N2**	*sp*Cbc1_RE_EcoRI	CGACGGAGCTCGAATTCTTATTTTTCCTCATTAGTTGCATCAG
**N3**	*sp*Cbc2_FW_NcoI	CACCATCACCATCACCCCATGGCATCAATAACAAGGTTAG
**N4**	*sp*Cbc2_RE_EcoRI	CGACGGAGCTCGAATTCTTAATTTTTTTTCCAACGATTGTATC
**N13**	*ct*Mtr4_FW_NcoI	GCTACCATGGATGACCTCTTTGCCGTC
**N14**	*ct*Mtr4_RE_BamHI	GCTAGGATCCTTACAGATACAAGCTGTTGAACGAG
**N59**	*sp*Ars2_215aa_RE	AATTGGTACCTTAGGATCCGTCTTGGTTCCATTTGGAAAGTTG
**N60**	*sp*Ars2_540aa_RE	AATTGGTACCTTAGGATCCTAAAATCTGTAAGGATACATACGACGTG
**N209**	*sp*Red1_345aa_NcoI_FW	TATACCATGGGTCTTACTATGTCTCGTTCAGAT
**N210**	*sp*Mtl1-dKOW-FW-Frg1-StuI	CTTTCAGAAGAAGATAGAGGCCTC
**N211**	*sp*Mtl1-dKOW-RE-Frg1-GA	GAACCAGAAGCTGATCCAGAACCGGTCATTACCTTTTGAATTTCTTCCC
**N212**	*sp*Mtl1-dKOW-FW-Frg2-GA	TTCTGGATCAGCTTCTGGTTCAGGTTCCTCTACGTTAAGTTTATCTCTTCGAAAAC
**N213**	*sp*Mtl1-dKOW-RE-Frg2-SacII	GTGTTGCGCAAGAGCCGCGG
**N214**	*sp*Mtl1-dArch-RE-Frg1-GA	CTCGAAGTTGTATAATCGCCTCAGATTCCATATTTTGGAACTGATAAAAG
**N215**	*sp*Mtl1-dArch-FW-Frg2-GA	GAGGCGATTATACAACTTCGAGAAC
**N241**	*sp*Mtl1-dArch-seq1-FW	TTAGACAAAGGGTTTTACGGCG
**N287**	*sp*Ars2_140aa_FW-NcoI	TATACCATGGTGGACGAGTCACAAGGAAGAG
**N288**	*sp*Ars2_516aa_RE-BamHI	TATACTCGAGTTAGGATCCATATCCATAGAGACATATAGCCACC
**N295**	*ct*Red1_1014aa_NcoI_FW	TATACCATGGCCTTGTCTTACTCCAGCCCTC
**N298**	*sp*Red1_577aa_NcoI_FW	TATACCATGGTCGATTATATCTCTCCATTTTACCGC
**N299**	*sp*Red1_712aa_BamHI-NotI-RE	TATAGCGGCCGCTTAGGATCCAATGGAAACCTTTGCAACAGGAATTGTC
**N320**	*ct*Red1-1030aa-NcoI-FW	TATACCATGGCTCCAGAGTTCACGCGGCTCG

**N321**	*ct*Red1-1040aa-NcoI-FW	TATACCATGGGTTGGAGGTCTC TGACTTACAG
**N322**	*ct*Red1-1091aa-XhoI-RE	TATACTCGAGTTAGGACAGTTC CAGGAGGATCCTTTC
**N326**	*sp*Mtl1-KOW-NcoI-FW	TATACCATGGGTCATCCTGATAA TTGTTTGCCTTATCTG
**N328**	*sp*Mtl1-Short-Arch-NcoI-FW	TATACCATGGGTATTCCCAATGA GACAGCTGTCAAG
**N329**	*sp*Mtl1-Short-Arch-BamHI-RE	TATAGGATCCTTAAGAATCCTTG TAGTAAGGGCTGTC
**N330**	*sp*Mtl1-Arch-NcoI-FW	TATACCATGGGTTTACCTTTAAT TAAAGAAGAACTTATGCAATT
**N331**	*sp*Mtl1-Arch-BamHI-RE	TATAGGATCCTTAAGTATTTGTA ATTTTCGTCGATATATCTTTTATC
**N332**	*sp*Red1-187aa-NcoI-FW	TATACCATGGCAAATTATTCCAA GACTACTAATCAAAAG
**N333**	*sp*Red1-187aa-BamHI-RE	TATAGGATCCTTATGCATCAGAA TTTTTAGATGTTGAAAG
**N334**	*sp*Red1-345aa-BamHI-RE	TATAGGATCCTTAACCATTTTCA CTATTTGAGGGGGG
**N350**	*sp*Mtl1-KOW-BamHI-RE	TATAGGATCCTTATTCATTCGAG TTCAACTCTCGTGG
**N353**	*sp*Iss10-180aa-XhoI-RE	TATACTCGAGTTAAGCTTGATTC CCATACAAAGATAAAG
**N354**	*sp*Iss10-180aa-NcoI-FW	TATACCATGGCTTTTAATCCTTC TTCAGTTCTTCCTAG
**N355**	*sp*Iss10-379aa-XhoI-RE	TATACTCGAGTTAAGCAGAAGG ACGAGGGTGAG
**N356**	*sp*Iss10-379aa-NcoI-FW	TATACCATGGCTGTCCTTTTAGA TAAACCGGTTTC
**N357**	*sp*Mmi1-344aa-XhoI-RE	TATACTCGAGTTAATTTTCGTCA AGAACAACTCGGG
**N358**	*sp*Mmi1-345aa-NcoI-FW	TATACCATGGGAATTTCACACC GCTCCCG
**N359**	*sp*Rmn1-326aa-XhoI-RE	TATACTCGAGTTATGCAAACTTC TTTGGTCGATTGTG
**N360**	*sp*Rmn1-326aa-NcoI-FW	TATACCATGGCATCCCATAAATC TCCCACTAC
**N361**	*sp*Ars2-296aa-XhoI-RE	TATACTCGAGTTAAGATTCAATA GTAAAATTTGAATCCAATTG
**N362**	*sp*Ars2-297aa-NcoI-FW	TATACCATGGAAAATCCAAAAAT TCCTACTCACACG
**N363**	*sp*Ars2-449aa-XhoI-RE	TATACTCGAGTTATTTTGCTTTA ATATCCACATTTTCAGG
**N364**	*sp*Ars2-450aa-NcoI-FW	TATACCATGGGGGCATTACCCG TTGAAGAT
**N377**	*sp*Red1-236aa-BamHI-RE	TATAGGATCCTTAGGAATCCAGT CCGAGCTTTAAAAA
**N378**	*sp*Red1-23aa-NcoI-FW	TATACCATGGATGAGAGTAATG ACTCTGACAAAG

**N379**	*sp*Red1-42aa-NcoI-FW	TATACCATGGTTATAGATCAATCAAATTCAGTTCCG
**N408**	5UTR-*sp*Red1-FW-GA-HindIII	CGGCCGCCAGCTGAAGCTTTACTAAATTACTGACTAGTCCATTAAAAGC
**N409**	5UTR-*sp*Red1-RE-GA	TTATTTACAGCTAAAACTGGGTTAGTAGTG
**N410**	*sp*Red1-CDS-FW-GA	AACCCAGTTTTAGCTGTAAATAAATGTCCCGGTCAATAAATCTCGATGAG
**N411**	*sp*Red1-CDS-RE-GA-AscI	GGCAAGCTAAACAGATCTGGCGCGCCTTAGGATCCAATGGAAACCTTTGCAACAGGAATTG
**N412**	3UTR-*sp*Red1-FW-GA-SacI	CGCTCGAAGGCTTTAACGAGCTCATTGAAACTGCCAAGCAACATCAAAC
**N413**	3UTR-*sp*Red1-RE-GA-SacI	ATCTGATATCATCGATGAATTCGAGCTGTGGTATTAATAGTTTGGCCTTTCATCC
**N414**	*sp*Red1-29-41aa-FW	CATGTCTGACAAAGAAGATGGCGAAATTAGCGAAGACGATCCTTAAG
**N415**	*sp*Red1-29-41aa-RE	GATCCTTAAGGATCGTCTTCGCTAATTTCGCCATCTTCTTTGTCAGA
**N416**	*sp*Red1-29-41aa(D33A-E35A)-FW	CATGTCTGACAAAGAAGCTGGCGCAATTAGCGAAGACGATCCTTAAG
**N417**	*sp*Red1-29-41aa(D33A-E35A)-RE	GATCCTTAAGGATCGTCTTCGCTAATTGCGCCAGCTTCTTTGTCAGA
**N422**	*sp*Mmi1-191aa-FW-NcoI	TATACCATGGGAAGTTCTCGTTATGTTGAAGAAGAAG
**N423**	*sp*Mmi1-191aa-RE-BamHI	TATAGGATCCTTATCCAACAACCACTGGGTCCGC
**N424**	*sp*Red1-scADH1-term-GA-BamHI-FW	TGTTGCAAAGGTTTCCATTGGATCCTAATCTCGAGGCGAATTTCTTATGATTTA
**N425**	*sp*Red1-scADH1-term-GA-BamHI-RE	AGATCTGGCGCGCCTTAGTCGATTACAACAGGTGTTGTCC
**N426**	*sp*Iss10-54aa-NcoI-FW	TATACCATGGGTTCTGTTAAACGGAAAGTCGAAATTGC
**N427**	*sp*Iss10-250aa-NcoI-FW	TATACCATGGGCCCGAGGGCAACTAACGTTGC
**N428**	*sp*Iss10-315aa-NcoI-FW	TATACCATGGGTAACGAAGAGCCGTCTGTCATTCTG
**N429**	*sp*Iss10-250aa-XhoI-RE	TATACTCGAGTTACGGAGGAGAAGGAGCTGACAC
**N430**	*sp*Iss10-315aa-XhoI-RE	TATACTCGAGTTAGTTTTTTACGGAGACAATTGAGTTTTTTTC

**N431**	*sp*Red1-537aa-seq-FW	TCAATAACCGAGGTTTCCGC
**N432**	*sp*Pla1-352aa-NcoI-FW	TATACCATGGACTTTTTTCATCG TTATAAGCATTATC
**N433**	*sp*Pla1-352aa-XhoI-RE	TATACTCGAGTTAGTCGTGTTTT TGAAACAAAGCTGAC
**N452**	*sp*Iss10-51aa-RE-XhoI	TATACTCGAGTTAATTTTTTAAA GATTTATGTAATCTATCTCTTAA
**N453**	*sp*Red1-653aa-RE-BamHI	TATAGGATCCTTATGATAAATCT TGTATAATTTCATCATCCG
**N464**	*ct*Red1-1091aa-C-6xHis-XhoI	TATACTCGAGTTAGTGATGGTG ATGGTGATGAGAACCGGACAGT TCCAGGAGGATCC
**N516**	*sp*Red1-216aa-BamHI-RE	TATAGGATCCTTAAATAAAATCG TTAAAGCGCACTCC
**N517**	*sp*Red1-217-236aa-Adaptor-NcoI-FW	CATGGCTGAGGGCATCGAGCCT TCGGTTGTTCATACTTTATTTTTA AAGCTCGGACTGGATTCCTAAG
**N518**	*sp*Red1-187-216aa-Adaptor-BamHI-RE	GATCCTTAGGAATCCAGTCCGA GCTTTAAAAATAAAGTATGAACA ACCGAAGGCTCGATGCCCTCAG C
**N519**	*ct*Red1-1040aa-FW-GA-BamHI	GTTCAACAGCTTGTATCTGGGC GGCTCCGGTTCCGGTTGGAGGT CTCTGACTTAC
**N520**	*ct*Red1-1091aa-RE-GA-BamHI	TGGTGGTGCTCGAGTTAGGATC CGGACAGTTCCAGGAGAATCCT TTCAT
**N521**	*sp*Mtr4-NcoI-FW-GA	AGAGGAGGACCTGGGTTCCATG GGTTTTGGTGGTGAGTTAGATG ATGC
**N522**	*sp*Mtr4-BamHI-RE-GA	CGCGGCCGCTCGAGTTAGGATC CCAAATATAGTGATGCACTAAAA ACAATATC
**N539**	*sp*Red1-288aa-NcoI-FW	TATACCATGGGCATATCTTTACC ACTTTTGAAGCAG
**N540**	*sp*Red1-287aa-BamHI-RE	TATAGGATCCTTAAAGATTTTTT CTTTCAGGAAGGAC
**N547**	*ct*Mtr4-short-Arch-NcoI-FW	TATACCATGGGCTCTGGACCCG ACGAGGCGCTCATCAAG
**N550**	*ct*Mtr4-short-Arch-BamHI-noStop-RE	TATAGGATCCCGAGTTGTGCAG CGGGTTG
**N551**	*ct*Mtr4-short-Arch-BamHI-RE	TATAGGATCCTTACGAGTTGTG CAGCGGGTTG
**N556**	*ct*Red1-1091-GA-RE	AGTCAGTGGTGGTGGTGGTGGT GGGATCCGGACAGTTCCAGGA GAATCCTTTCATC
**N557**	*ct*Red1-1091-STOP-GA-RE	AGTCAGTGGTGGTGGTGGTGGT GGTTAGGATCCGGACAGTTCCA GGAGAATCCTTTCATC

**N558**	*ct*Red1-1014-GGSGGS-GA-FW	CCCGCTGCACAACTCGGGTGGT TCTGGCGGTTCCGCCTTGTCTT ACTCCAGCCC
**N577**	*ct*Mtr4-Arch-632-NcoI-FW	TATACCATGGGTAGCATCCCCC AGCTAGAGC
**N578**	*ct*Mtr4-Arch-897-BamHI-RE	TATATTAGGATCCGGAGTGCGC ACGCGAGATC
**N579**	*ct*Mtr4-KOW-dLoop1-FW-QC	GAGGGTAGCGGTTCTTACGTCG TCGAGGTACTCCTCC
**N580**	*ct*Mtr4-KOW-dLoop1-RE-QC	ACGTAAGAACCGCTACCCTCGT AGAAGTTGATGATGACGCC
**N581**	*ct*Mtr4-KOW-dLoop2-FW-QC	GGCAGCGGTAGCGGTTCTGGC CGCTGGGAGGTCGTG
**N582**	*ct*Mtr4-KOW-dLoop2-RE-QC	AGAACCGCTACCGCTGCCGGAT AGGCGGAGGAGTACCTC
**N583**	*sp*Red1-29-41aa-NoSTOP-FW-XhoI	CATGTCTGACAAAGAAGATGGC GAAATTAGCGAAGACGATCCTC
**N584**	*sp*Red1-29-41aa-NoSTOP-RE-XhoI	TCGAGAGGATCGTCTTCGCTAA TTTCGCCATCTTCTTTGTCAGA
**N602**	*ct*Red1-F1024R-FW-QC	CCAGCCCTCTACGCAGGTTCCG GAACTTCCGATTC
**N603**	*ct*Red1-F1024R-RE-QC	GAATCGGAAGTTCCGGAACCTG CGTAGAGGGCTGG
**N604**	*ct*Red1-I1050R-FW-QC	CAGCAGGAGAGATCCTGACAAA GAGATGTGCC
**N605**	*ct*Red1-I1050R-RE-QC	GTCAGGATCTCTCCTGCTGCTG TAAGTCAGAGAC
**N606**	*ct*Red1-S1043R-T1045R-FW-QC	TGGAGGAGACTGAGATACAGCA GCAGGATTGATCCTGAC
**N607**	*ct*Red1-S1043R-T1045R-FW-QC	CTGTATCTCAGTCTCCTCCAACC ACCGGCCACG
**N608**	*ct*Red1-E1060R-FW-QC	CCCATATAGACTGGAGGGGACA CAGTGCC
**N609**	*ct*Red1-E1060R-RE-QC	CCCCTCCAGTCTATATGGGCAC ATCTCTTTGTCAGG
**N610**	*sp*Red1_F586R-FW-QC	ATTTTACCGCAGAAAATCTTATC GATTTAATCAACAATTTGTTGAG
**N611**	*sp*Red1_F586R-RE-QC	CGATAAGATTTTCTGCGGTAAAA TGGAGAGATATAATCGAC
**N612**	*sp*Red1_S605R_T607R-FW-QC	TATCGGAGGCTTAGGTACAGCA ATAAGATAGAGCCGATGAA
**N613**	*sp*Red1_S605R_T607R-RE-QC	CTGTACCTAAGCCTCCGATATTT CAACGGTACTCGCTC
**N614**	*sp*Red1_I612R-FW-QC	GCAATAAGAGAGAGCCGATGAA AGTTTTCTGTAAATAC
**N615**	*sp*Red1_I612R-RE-QC	CATCGGCTCTCTCTTATTGCTGT AGGTAAGCGACCG
**N616**	*sp*Red1_E622R-FW-QC	TGTAAATACAGAACCACTGGTG GTGTTTGTAATGATG

**N617**	*sp*Red1_E622R-RE-QC	CACCAGTGGTTCTGTATTTACAG AAAACTTTCATCGGCTC
**N618**	*sp*Red1_259aa-FW-GA-BamHI	GAAGAACGGCCGAAAGCTGGT GGTTCTGGCGGTTCCCGTAAGA TTGACAGTAATCTTAGTG
**N619**	*sp*Red1_345aa-RE-GA-BamHI	GTCAGTGGTGGTGGTGGTGGT GGGATCCATTTTCACTATTTGAG GGGG
**N620**	*sp*Red1_288aa-FW-GA-BamHI	GAAGAACGGCCGAAAGCTGGT GGTTCTGGCGGTTCCATATCTTT ACCACTTTTGAAGCAGG
**N621**	*sp*Red1_321aa-RE-GA-BamHI	GTCAGTGGTGGTGGTGGTGGT GGGATCCATCATCATCGGAATC AAATTCAATAAC
**N624**	*sp*Red1-236aa-NoStop-BamHI-RE	TATAGGATCCGGAATCCAGTCC GAGCTTTAAAAA
**N625**	*sp*Red1-345aa-NoStop-BamHI-RE	TATAGGATCCACCATTTTCACTA TTTGAGGGGGG
**N652**	*sp*Red1_F586A_K587D_Frg1_RE-GA	GATAAGAATCAGCGCGGTAAAA TGGAGAGATATAATCG
**N653**	*sp*Red1_F586A_K587D_Frg2_FW-GA	ATTTTACCGCGCTGATTCTTATC GATTTAATCAACAATTTGTTGAG
**N654**	*sp*Red1_I641R_Frg1_RE-GA	CGTCATTTTTCTGTCGCGAAAAT GCGACGCC
**N655**	*sp*Red1_I641R_Frg2_FW-GA	ATTTTCGCGACAGAAAAATGAC GGATGATGAAATTATACAAG
**N656**	*sp*Red1_S581D_F583D_Frg1_RE-GA	GCGGTATTCTGGATCGATATAAT CGACAAGCGGTACC
**N657**	*sp*Red1_S581D_F583D_Frg2_FW-GA	TATATCGATCCAGAATACCGCG CTGATTCTTATCGAT
**N658**	*sp*Red1_T623D_T623R_Frg1_RE-GA	CACCACCACGATCTTCGTATTTA CAGAAAACTTTCATCGG
**N659**	*sp*Red1_T623D_T623R_Frg2_FW-GA	TACGAAGATCGTGGTGGTGTTT GTAATGATGACCATTG
**N660**	*sp*Red1-576aa-BamHI-STOP-XhoI-RE	ATATCTCGAGTTAGGATCCAAG CGGTACCTTATCCTCAAAATC
**N661**	*sp*Red1_5AA-mutant_dete*ct*_RE	ATCAGCGCGGTATTCTGGATC
**N662**	*sp*Red1-602aa-NcoI-FW	TATACCATGGGCAAATATCGGT CGCTTACCTACAGC
**N663**	*sp*Mlt1-SA-F666E-FW-QC	AAAAGAGAAGAAGATCCAAACA CTCGTGAACAAGTTG
**N664**	*sp*Mlt1-SA-F666E-RE-QC	AGTGTTTGGATCTTCTTCTCTTT TTATGACATTGACAAGTACAC
**N665**	*sp*Mlt1-SA-I652E-FW-QC	GGGGTATTGAATTTCCTTGGGG TGTACTTGTCAATG
**N666**	*sp*Mlt1-SA-I652E-RE-QC	CCAAGGAAATTCAATACCCCCT AATTTTATTTGTATAAGTCG
**N683**	*sp*Red1_F586A_Frg1_RE-GA	CGATAAGATTTGGCGCGGTAAA ATGGAGAGATATAATC

**N684**	*sp*Red1_F586A_Frg2_FW-GA	TACCGCGCCAAATCTTATCGATT TAATCAACAATTTGTTG
**N685**	*sp*Red1_K587D_Frg1_RE-GA	AATCGATAAGAGTCAAAGCGGT AAAATGGAGAGATATAATC
**N686**	*sp*Red1_K587D_Frg2_FW-GA	ACCGCTTTGACTCTTATCGATTT AATCAACAATTTGTTG
**N687**	*sp*Red1_F586A_mut_check_RE	GTTGATTAAATCGATAAGATTTG GC
**N688**	*sp*Red1_F586F_wt_check_RE	ATTGTTGATTAAATCGATAAGAT TTAAA
**N689**	*sp*Red1_K587D_mut_check_RE	ATTGTTGATTAAATCGATAAGAG TC
**N690**	*sp*Red1_K587K_wt_check_RE	CAAATTGTTGATTAAATCGATAA GATTT
**N696**	*sp*Mtl1_I730R-frg1-GA-RE	ATAAATACGTCTAGAAGAAATGT TACAAACAG
**N697**	*sp*Mtl1_I730R-frg2-GA-FW	TAACATTTCTTCTAGACGTATTT ATATGCC
**N698**	*sp*Mtl1_F758R-frg1-GA-RE	GTATTTCCTTTCTTTCTTCTATAA CCTCATTTAC
**N699**	*sp*Mtl1_F758R-frg2-GA-FW	GTTATAGAAGAAAGAAAGGAAA TACCTTATTTAG
**N700**	*sp*Mtl1_L781R-frg1-GA-RE	CAAGTTTTCGTCTAGATAAACTT AACGTAG
**N701**	*sp*Mtl1_L781R-frg2-GA-FW	TTAAGTTTATCTAGACGAAAACT TGAAATTC
**N702**	*sp*Mtl1_L784R_E788R-frg1-GA-RE	GGTCTAAGAATTTCTCTTTTTCG AAGAGATAAACTTAACG
**N703**	*sp*Mtl1_L784R_E788R-frg2-GA-FW	CGAAAAAGAGAAATTCTTAGAC CCAAACTCTTCGACAGCC
**N704**	*sp*Mtl1-dSA-frg1-GA-RE	CCAGAACCTGATCCAGAACCCT CATTGGGAATATTAATTGAAGTC
**N705**	*sp*Mtl1-dSA-frg2-GA-FW	GTTCTGGATCAGGTTCTGGTTC AGATTCTAAACATAGGGCCGAA TATC
**N725**	*sp*Mtl1_E788R-frg1-GA-RE	GAAATTCTTAGACCCAAACTCTT CGACAGCCC
**N726**	*sp*Mtl1_E788R-frg2-GA-FW	GAGTTTGGGTCTAAGAATTTCAA GTTTTCGAAGAG
**N/A**	Red1-qPCR-FW	CGACCATTGGCTTTATCATCC
**N/A**	Red1-qPCR-RE	TTCAAACAACTGACTCACAGC
**N/A**	GAPDH-qPCR-FW	AACATCATCCCCTCCTCCAC
**N/A**	GAPDH-qPCR-RE	GCCTTGATGTCCTCGTAGTTG

Supplementary Table 3. Plasmids used in this study

pND #	Plasmid	Selection	References
***E. coli* expression plasmids**			
72	pET_His-EYFP	Kan	Gunter Stier
76	pET_His_1a-EYFP	Kan	Gunter Stier
81	pET_GST_1a-EYFP	Kan	Gunter Stier
82	pET_MBP_1a-EYFP	Kan	Gunter Stier
92	pET_His-*ct*Mtr4_1-1097aa	Kan	this study
434	pET_GST_1a-Stop-control	Kan	this study
565	pET_GST_1a-*sp*Red1_29-41aa	Kan	this study
566	pET_GST_1a-*sp*Red1_29-41aa-D33A, E35A	Kan	this study
644	pET21d-GST_*a*-*ct*Red1_1040-1091aa	Amp	this study
646	pET21d-ZZ_*a*-*ct*Red1_1040-1091aa	Amp	this study
689	pET21d_MBP-*sp*Iss10_1-51aa	Amp	this study
690	pET_GST_1a-*sp*Iss10_1-51aa	Kan	this study
699	pET_GST_1a-*sp*Red1_1-236aa	Kan	this study
733	pET_MBP_1a-*sp*Red1_187-236aa	Kan	this study
828	pET21d_T4L_xtal-*ct*Red1_1014-1091aa	Amp	this study
865	pET24d-*ct*Mtr4_654-866aa	Kan	this study
868	pET24dC-6xHis-*ct*Mtr4-*ct*Red1_654-865 | 1014-1091aa	Kan	this study
887	pET24d-*ct*Mtr4_654-866aa-dLoop1	Kan	this study
888	pET24d-*ct*Mtr4_654-866aa-dLoop2	Kan	this study
917	pET24d-*ct*Mtr4_654-866aa-dLoop1+2	Kan	this study
919	pET21d-Gb1_*a*-*ct*Red1_1014-1091aa	Amp	this study
936	pET21d_T4L_xtal-*ct*Red1_1014-1091aa-F1024R	Amp	this study
937	pET21d_T4L_xtal-*ct*Red1_1014-1091aa-I1050R	Amp	this study
938	pET21d_T4L_xtal-c*t*Red1_1014-1091aa-S1043R,T1045R	Amp	this study
939	pET21d_T4L_xtal-*ct*Red1_1014-1091aa-E1060R	Amp	this study
1003	pET_His_1a-*ct*Mtr4_short arch	Kan	this study
**Y2H/Y3H plasmids**			
333	pGBKT7	Kan	this study
334	pGADT7	Amp	this study
367	pGBKT7-*sp*Red1_1-712aa	Kan	this study
368	pGADT7-*sp*Red1_1-712aa	Amp	this study
369	pGBKT7-*sp*Mtl1_1-1030aa	Kan	this study
370	pGADT7-*sp*Mtl1_1-1030aa	Amp	this study
371	pGBKT7-*sp*Red1_345-712aa	Kan	this study
376	pGBKT7-*sp*Ars2_1-609aa	Kan	this study

377	pGBKT7-*ct*Red1_1014-1055aa	Kan	this study
378	pGBKT7-*ct*Red1_1014-1152aa	Kan	this study
379	pGBKT7-*ct*Red1_676-1055aa	Kan	this study
380	pGBKT7-*sp*Red1_577-712aa	Kan	this study
387	pG4ADC111_mod	Amp	Modified from[12]
388	pG4ADHAN111_mod	Amp	Modified from[12]
389	pG4BDN22_mod	Amp	Modified from[12]
390	pG4BDC22_mod	Amp	Modified from[12]
396	pGBKT7-*sp*Mtl1_dArch	Kan	this study
397	pGBKT7-*sp*Mtl1_dKOW	Kan	this study
398	pGADT7-*sp*Red1_345-712aa	Amp	this study
399	pGADT7-*sp*Red1_577-712aa	Amp	this study
400	pRS426	Amp	Tomlin et al., 2011 [13]
411	pGBKT7-*sp*Red5_1-376aa	Kan	this study
412	pGBKT7-*sp*Iss10_1-551aa	Kan	this study
413	pGBKT7-*sp*Mmi1_1-488aa	Kan	this study
414	pGBKT7-*sp*Pab2_1-166aa	Kan	this study
415	pGBKT7-*sp*Rmn1_1-590aa	Kan	this study
420	pG4BDN-*sp*Nrl1_1-972aa	Amp	this study
435	pGADT7-*sp*Red5_1-376aa	Amp	this study
436	pGADT7-*sp*Iss10_1-551aa	Amp	this study
437	pGADT7-*sp*Mmi1_1-488aa	Amp	this study
438	pGADT7-*sp*Pab2_1-166aa	Amp	this study
439	pGADT7-*sp*Rmn1_1-590aa	Amp	this study
444	pGBKT7-*sp*Ars2_140-516aa	Kan	this study
446	pGADT7-*sp*Red1_1-187aa	Amp	this study
447	pGADT7-*sp*Red1_1-345aa	Amp	this study
448	pGADT7-*sp*Red1_187-345aa	Amp	this study
449	pGADT7-*sp*Red1_187-712aa	Amp	this study
450	pGBKT7-*sp*Mtl1_short arch	Kan	this study
451	pGBKT7-*sp*Mtl1_Arch	Kan	this study
452	pGBKT7-*sp*Ars2_1-516aa	Kan	this study
453	pGBKT7-*sp*Ars2_79-609aa	Kan	this study
454	pGBKT7-*sp*Ars2_140-609aa	Kan	this study
469	pGBKT7-*sp*Pla1_1-566aa	Kan	this study
473	pRS426-ADH1	Amp	this study
474	pGBKT7-*sp*Iss10_1-180aa	Kan	this study
475	pGBKT7-*sp*Iss10_1-379aa	Kan	this study
476	pGBKT7-*sp*Iss10_180-379aa	Kan	this study
477	pGBKT7-*sp*Iss10_180-551aa	Kan	this study
478	pGBKT7-*sp*Iss10_379-551aa	Kan	this study
479	pGADT7-*sp*Mmi1_1-344aa	Amp	this study
480	pGADT7-*sp*Mmi1_345-488aa	Amp	this study

481	pGBKT7-*sp*Rmn1_1-326aa	Kan	this study
482	pGBKT7-*sp*Rmn1_326-590aa	Kan	this study
483	pGADT7-*sp*Rmn1_1-326aa	Amp	this study
484	pGADT7-*sp*Rmn1_326-590aa	Amp	this study
485	pGBKT7-*sp*Ars2_140-296aa	Kan	this study
486	pGBKT7-*sp*Ars2_140-449aa	Kan	this study
487	pGBKT7-*sp*Ars2_297-516aa	Kan	this study
488	pGBKT7-*sp*Ars2_450-516aa	Kan	this study
490	pGBKT7-*sp*Pab2_1-136aa	Kan	this study
494	pGADT7-*sp*Red1_187-236aa	Amp	this study
495	pGADT7-*sp*Red1_24-712aa	Amp	this study
496	pGADT7-*sp*Red1_42-712aa	Amp	this study
497	pRS426-ADH1-*sp*Red1_1-712aa	Amp	this study
498	pRS426-ADH1-*sp*Red5_1-376aa	Amp	this study
499	pRS426-ADH1-*sp*Iss10_1-551aa	Amp	this study
500	pRS426-ADH1-*sp*Mmi1_1-488aa	Amp	this study
501	pRS426-ADH1-*sp*Pab2_1-166aa	Amp	this study
502	pRS426-ADH1-*sp*Rmn1_1-590aa	Amp	this study
507	pG4BDN-*sp*Mtl1_1-1030aa	Amp	this study
508	pG4ADHAN-*sp*Mtl1_1-1030aa	Amp	this study
509	pG4ADHAN-*sp*Red1_1-712aa	Amp	this study
526	pG4ADHAN-*sp*Mtl1_dKOW	Amp	this study
527	pG4ADHAN-*sp*Mtl1_dArch	Amp	this study
528	pG4ADHAN-*sp*Mtl1_short arch	Amp	this study
529	pG4ADHAN-*sp*Mtl1_Arch	Amp	this study
542	pGADT7-*sp*Red1_29-41aa	Amp	this study
543	pGADT7-*sp*Red1_29-41aa-D33A, E35A	Amp	this study
548	pGADT7-*sp*Mmi1_1-191aa	Amp	this study
549	pGADT7-*sp*Mmi1_191-344aa	Amp	this study
550	pG4ADHAN-*sp*Mtl1_short arch-dKOW	Amp	this study
551	pG4ADHAN-*sp*Mtl1_Arch-dKOW	Amp	this study
556	pGBKT7-*sp*Iss10_1-250aa	Kan	this study
557	pGBKT7-*sp*Iss10_1-315aa	Kan	this study
558	pGBKT7-*sp*Iss10_54-379aa	Kan	this study
559	pGBKT7-*sp*Iss10_250-551aa	Kan	this study
560	pGBKT7-*sp*Iss10_315-551aa	Kan	this study
561	pGBKT7-*sp*Pla1_1-352aa	Kan	this study
562	pGBKT7-*sp*Pla1_352-566aa	Kan	this study
563	pGBKT7-*sp*Ars2_440-516aa	Kan	this study
574	pG4BDN-*sp*Cbc1_1-780aa	Amp	this study
575	pG4ADHAN-*sp*Cbc1_1-780aa	Amp	this study
576	pRS426-ADH1-*sp*Cbc1_1-780aa	Amp	this study
577	pG4BDN-*sp*Cbc2_1-182aa	Amp	this study

578	pG4ADHAN-*sp*Cbc2_1-182aa	Amp	this study
579	pRS426-ADH1-*sp*Cbc2_1-182aa	Amp	this study
580	pG4BDN-*sp*Ars2_1-609aa	Amp	this study
581	pG4ADHAN-*sp*Ars2_1-609aa	Amp	this study
582	pRS426-ADH1-*sp*Ars2_1-609aa	Amp	this study
589	pG4BDN-*sp*Ntr1_1-797aa	Amp	this study
668	pG4BDN-*sp*Mtl1_short arch	Amp	this study
669	pG4BDN-*sp*Mtl1_Arch	Amp	this study
670	pG4ADC-*sp*Cbc2_1-182aa	Amp	this study
672	pG4BDC-*sp*Cbc2_1-182aa	Amp	this study
714	pGADT7-*sp*Red1_50-345aa	Amp	this study
715	pGADT7-*sp*Red1_100-345aa	Amp	this study
760	pGBKT7-*sp*Iss10_1-51aa	Kan	this study
761	pGADT7-*sp*Red1_187-216aa	Amp	this study
762	pGADT7-*sp*Red1_217-236aa	Amp	this study
776	pG4ADHAN-*sp*Red1_1-236aa	Amp	this study
779	pG4ADHAN-*sp*Red1_240-712aa	Amp	this study
1004	pGADT7-*sp*Red1_1-712aa-I612R	Amp	this study
1016	pGADT7-*sp*Red1_1-712aa-F585A_K586D	Amp	this study
1017	pGADT7-*sp*Red1_1-712aa-I641R	Amp	this study
1018	pGADT7-*sp*Red1_1-712aa-F585A_K586D_I612R	Amp	this study
1019	pGADT7-*sp*Red1_1-712aa-I612R_I641R	Amp	this study
1041	pGADT7-*sp*Red1_1-712aa-S581D_F583E_F586A_R587D_I612R	Amp	this study
1042	pGADT7-*sp*Red1_1-712aa-F586A_R587D_I612R_T623D_T624R	Amp	this study
1044	pG4ADHAN-*sp*Red1_1-530aa	Amp	this study
1045	pG4ADHAN-*sp*Red1_1-576aa	Amp	this study
1046	pG4ADC-*sp*Red1_1-576aa	Amp	this study
1061	pG4BDN-*sp*Mtl1_1-1030aa-I730R	Amp	this study
1062	pG4BDN-*sp*Mtl1_1-1030aa-F758R	Amp	this study
1063	pG4BDN-*sp*Mtl1_1-1030aa-L781R	Amp	this study
1064	pG4BDN-*sp*Mtl1_1-1030aa-L784R_E788R	Amp	this study
1065	pG4BDN-*sp*Mtl1_1-1030aa-dSA	Amp	this study

S. pombe integration plasmids

1039	pFab6-Red1-gDNA_WT	Amp	this study
1057	pFab6-Red1-gDNA_S581D_F583E_F586A_R587D_I612R	Amp	this study
1068	pFab6-Nat2-Mtl1-L784R_E788R	Amp	this study

Supplementary References

1. Lee, N. N. *et al.* Mtr4-like protein coordinates nuclear RNA processing for heterochromatin assembly and for telomere maintenance. *Cell* **155**, 1061–1074 (2013).
2. Zhou, Y. *et al.* The fission yeast MTREC complex targets CUTs and unspliced pre-mRNAs to the nuclear exosome. *Nat. Commun.* **6**, (2015).
3. Emsley, P., Lohkamp, B., Scott, W. G. & Cowtan, K. Features and development of Coot. *Acta Crystallogr. D Biol. Crystallogr.* **66**, 486–501 (2010).
4. Ashkenazy, H., Erez, E., Martz, E., Pupko, T. & Ben-Tal, N. ConSurf 2010: calculating evolutionary conservation in sequence and structure of proteins and nucleic acids. *Nucleic Acids Res.* **38**, W529-533 (2010).
5. Krissinel, E. & Henrick, K. Inference of macromolecular assemblies from crystalline state. *J. Mol. Biol.* **372**, 774–797 (2007).
6. Falk, S. *et al.* The molecular architecture of the TRAMP complex reveals the organization and interplay of its two catalytic activities. *Mol. Cell* **55**, 856–867 (2014).
7. Lingaraju, M. *et al.* The MTR4 helicase recruits nuclear adaptors of the human RNA exosome using distinct arch-interacting motifs. *Nat. Commun.* **10**, 3393 (2019).
8. Falk, S. *et al.* Structural insights into the interaction of the nuclear exosome helicase Mtr4 with the preribosomal protein Nop53. *RNA N. Y. N* **23**, 1780–1787 (2017).

9. Wang, J. *et al.* NRDE2 negatively regulates exosome functions by inhibiting MTR4 recruitment and exosome interaction. *Genes Dev.* **33**, 536–549 (2019).
10. Sugiyama, T. & Sugioka-Sugiyama, R. Red1 promotes the elimination of meiosis-specific mRNAs in vegetatively growing fission yeast. *EMBO J.* **30**, 1027–1039 (2011).
11. James, P., Halladay, J. & Craig, E. A. Genomic libraries and a host strain designed for highly efficient two-hybrid selection in yeast. *Genetics* **144**, 1425–1436 (1996).
12. Thoms, M. *et al.* The Exosome Is Recruited to RNA Substrates through Specific Adaptor Proteins. *Cell* **162**, 1029–1038 (2015).
13. Tomlin, G. C., Wixon, J. L., Bolotin-Fukuhara, M. & Oliver, S. G. A new family of yeast vectors and S288C-derived strains for the systematic analysis of gene function. *Yeast Chichester Engl.* **18**, 563–575 (2001).
