# Supplementary material for: The zinc-finger protein Red1 orchestrates MTREC submodules and binds the Mtl1 helicase arch domain
Source: Nat Commun. 2021 Jun 8;12:3456. doi: 10.1038/s41467-021-23565-3 (PMC8187409; doi:10.1038/s41467-021-23565-3)
Supplement: Supplementary file 2 — Reporting summary [file 41467_2021_23565_MOESM2_ESM.pdf]

nature research

Corresponding author(s): Irmgard Sinning, Tamás Fischer

Last updated by author(s): May 4, 2021

Reporting Summary

Nature Research wishes to improve the reproducibility of the work that we publish. This form provides structure for consistency and transparency in reporting. For further information on Nature Research policies, see Authors & Referees and the Editorial Policy Checklist .

Statistics

For all statistical analyses, confirm that the following items are present in the figure legend, table legend, main text, or Methods section.

n/a	Confirmed	
☐	☒	The exact sample size (n) for each experimental group/condition, given as a discrete number and unit of measurement
☐	☒	A statement on whether measurements were taken from distinct samples or whether the same sample was measured repeatedly
☒	☐	The statistical test(s) used AND whether they are one- or two-sided<br>*Only common tests should be described solely by name; describe more complex techniques in the Methods section.*
☒	☐	A description of all covariates tested
☒	☐	A description of any assumptions or corrections, such as tests of normality and adjustment for multiple comparisons
☐	☒	A full description of the statistical parameters including central tendency (e.g. means) or other basic estimates (e.g. regression coefficient) AND variation (e.g. standard deviation) or associated estimates of uncertainty (e.g. confidence intervals)
☒	☐	For null hypothesis testing, the test statistic (e.g. F, t, r) with confidence intervals, effect sizes, degrees of freedom and P value noted<br>*Give P values as exact values whenever suitable.*
☒	☐	For Bayesian analysis, information on the choice of priors and Markov chain Monte Carlo settings
☒	☐	For hierarchical and complex designs, identification of the appropriate level for tests and full reporting of outcomes
☒	☐	Estimates of effect sizes (e.g. Cohen's d, Pearson's r), indicating how they were calculated

Our web collection on statistics for biologists contains articles on many of the points above.

Software and code

Policy information about availability of computer code

Data collection	MicroCal ITC Control Software (version 1.21) for ITC, qPCR data were acquired with QuantStudio 12k Flex software. ASTRA 6.1 software was used for SEC-MALS. mxCuBE software was used at European Synchrotron Radiation Facility (ESRF, Grenoble) at beamline ID29 for X-ray data collection.
Data analysis	ITC data were analyzed with MicroCal ITC Analysis Software (version 1.21). ASTRA 6.1 software was used to analyze the SEC-MALS experiments. qPCR data were analyzed with Microsoft Excel using delta delta Ct method.<br>Data were processed with XDS, and Phases were obtained by SHELX. Molecular models were built with COOT and refined using Phenix and Refmac5. Structures and electron densities were visualised and analyzed with and PYMOL (Schrödinger, LLC). Sequence alignments were performed using Clustal Omega and visualized with ESPript servers.

For manuscripts utilizing custom algorithms or software that are central to the research but not yet described in published literature, software must be made available to editors/reviewers. We strongly encourage code deposition in a community repository (e.g. GitHub). See the Nature Research guidelines for submitting code & software for further information.

Data

Policy information about availability of data

All manuscripts must include a data availability statement. This statement should provide the following information, where applicable:

- Accession codes, unique identifiers, or web links for publicly available datasets
- A list of figures that have associated raw data
- A description of any restrictions on data availability

Accession codes: atomic coordinates for the ctMtr4-ctRed1 minimal complex were deposited at the Protein Data Bank under accession codes 6YGU (single chain) and 6YFV (native complex).
The PDB datasets 2XGJ, 4U4C, 6RO1, 5OOQ and 6IEH have been used in this study.
Protein sequences from Uniprot database with the following IDs were used in this study: G0RZ64, O13799, P42285, O14232, P47047, G0S1V1, Q9UTR8, V5IR63,

B2RT41, O60293, G0SE05, O14253, G0S4F4, Q9P383, G0SBQ9, O94326, Q9BXP5-4, G0S5V0, Q9USP9, G0RZM1, O74823, G0S9J4, O14327, G0S6X0, Q10295.
The data underlying Fig. 6d as well as Supplementary Fig. 13b and the uncropped images of the gel shown in Figs. 3b and 5a, Supplementary Figs. 1f, 2c and 12b are provided as a Source Data file.
Other data that support the findings of this study are available from the corresponding authors on reasonable request.

Field-specific reporting

Please select the one below that is the best fit for your research. If you are not sure, read the appropriate sections before making your selection.

☒ Life sciences ☐ Behavioural & social sciences ☐ Ecological, evolutionary & environmental sciences

For a reference copy of the document with all sections, see nature.com/documents/nr-reporting-summary-flat.pdf

Life sciences study design

All studies must disclose on these points even when the disclosure is negative.

Sample size	n = 2
Data exclusions	The first data point from the ITC experiments was excluded as this is a normal well-accepted practice in analysis ITC data.
Replication	qPCR experiments were performed in technical triplicates and two biological replicates.
Randomization	This study did not involve experimental groups, therefore randomization was not performed.
Blinding	No experiments which required blinding were performed in the current study.

Reporting for specific materials, systems and methods

We require information from authors about some types of materials, experimental systems and methods used in many studies. Here, indicate whether each material, system or method listed is relevant to your study. If you are not sure if a list item applies to your research, read the appropriate section before selecting a response.

Materials & experimental systems

n/a	Involved in the study
☒	☐ Antibodies
☒	☐ Eukaryotic cell lines
☒	☐ Palaeontology
☒	☐ Animals and other organisms
☒	☐ Human research participants
☒	☐ Clinical data

Methods

n/a	Involved in the study
☒	☐ ChIP-seq
☒	☐ Flow cytometry
☒	☐ MRI-based neuroimaging
